# Protocolized REDUction of Non‐Resuscitation Fluids in SEptic Shock Patients. A Protocol for the REDUSE Randomized Clinical Trial

**DOI:** 10.1111/aas.70095

**Published:** 2025-07-16

**Authors:** Peter Bentzer, Anja Lindén, Markus H. Olsen, Gisela Lilja, Jane Fisher, Fredrik Sjövall, Thomas Kander, Maria Lengquist, Line Samuelsson, Johan Undén, Ewa Palmnäs, Jonatan Oras, Maria Cronhjort, Anca Balintescu, Alicia Lind, Björn Ahlström, Maria Meirik, Johanna Savilampi, Pirkka Pekkarinen, Anna Berggren, Nicklas Oscarsson, Mohammed Said, Markus Castegren, Susanne Faria, Linn Hemberg, Adam Linder, Miklos Lipcsey, Marcus B. Skrifvars, Matt P. Wise, Niklas Nielsen, Janus C. Jakobsen

**Affiliations:** ^1^ Anesthesiology and Intensive Care, Department of Clinical Sciences Malmö Lund University Lund Sweden; ^2^ Department of Intensive and Perioperative Care Skåne University Hospital Malmö Sweden; ^3^ Department of Anaesthesiology and Intensive Care Helsingborg Hospital Helsingborg Sweden; ^4^ Copenhagen Trial Unit Centre for Clinical Intervention Research, The Capital Region, Copenhagen University Hospital Copenhagen Denmark; ^5^ Department of Neuroanaesthesiology The Neuroscience Centre, Copenhagen University Hospital Copenhagen Denmark; ^6^ Neurology, Department of Clinical Sciences Lund Lund University Lund Sweden; ^7^ Neurology Department Skåne University Hospital Lund Sweden; ^8^ Anaesthesiology and Intensive Care, Department of Clinical Sciences Lund Lund University Lund Sweden; ^9^ AdvanSci Research Solutions Lund Sweden; ^10^ Department of Intensive and Perioperative Care Skåne University Hospital Lund Sweden; ^11^ Department of Anaesthesiology and Intensive Care Östersund Hospital Östersund Sweden; ^12^ Department of Operation and Intensive Care Hallands Hospital Halmstad Halmstad Sweden; ^13^ Department of Anaesthesiology and Intensive Care Medicine Sahlgrenska Academy, University of Gothenburg Gothenburg Sweden; ^14^ Karolinska Institutet, Department of Clinical Sciences Danderyd Hospital Stockholm Sweden; ^15^ Department of Clinical Science and Education Section of Perioperative and Intensive Care, Karolinska Institute Södersjukhuset Stockholm Sweden; ^16^ Department of Diagnostics and Intervention Umeå University and Umeå Centre for Microbial Research, Umeå University Umeå Sweden; ^17^ Department of Anaesthesiology and Intensive Care Falun Hospital Falun Sweden; ^18^ Anaesthesiology and Intensive Care Medicine, Department of Surgical Sciences Uppsala University Uppsala Sweden; ^19^ Department of Operation and Intensive Care Hallands Hospital Varberg Varberg Sweden; ^20^ Department of Anaesthesia and Intensive Care, Faculty of Medicine and Health Örebro University Örebro Sweden; ^21^ Department of Anaesthesia and Intensive Care Helsinki University Hospital and University of Helsinki Helsinki Finland; ^22^ Department of Anaesthesia and Intensive Care Norrtälje Hospital Norrtälje Sweden; ^23^ Division of Pediatric Oncology, Department of Women and Children's Health Karolinska Institutet Stockholm Sweden; ^24^ Department of Anaesthesia and Intensive Care Södertälje Hospital Södertälje Sweden; ^25^ Centre for Clinical Research Sörmland Uppsala University Uppsala Sweden; ^26^ Department of Clinical Sciences Karolinska Institutet Stockholm Sweden; ^27^ Infectious Diseases, Department of Clinical Sciences Lund Lund University Lund Sweden; ^28^ Adult Critical Care, University Hospital of Wales Cardiff UK; ^29^ Department of Regional Health Research, The Faculty of Health Sciences University of Southern Denmark Odense Denmark

## Abstract

In septic shock, administration of large fluid volumes is associated with poor outcomes. Recent evidence shows that non‐resuscitation fluids are the major modifiable source of fluids for patients with septic shock in intensive care units (ICUs). This clinical trial is designed to test the hypothesis that restrictive administration of non‐resuscitation fluids improves outcomes compared to usual care. Adult patients admitted to ICUs with septic shock will be randomly assigned within 12 h of admission to receive protocolized restrictive administration of non‐resuscitation fluids or usual care. The primary outcome is all‐cause mortality at 90 days. Secondary outcomes are complications during ICU stay up to 90 days (defined as any acute kidney injury or cerebral, coronary, intestinal, or limb ischemia), mechanical ventilation free days within 90 days, and for survivors cognitive function (by the Montreal Cognitive Assessment [MOCA‐BLIND]) and Health‐Related Quality of Life (by the EQ Visual Analogue Scale [EQ‐VAS]), both at 6 months. In addition, the climate impact of the interventions will be assessed. To detect an absolute reduction in mortality of 7.5%, with an alpha of 5% and a power of 90%, we aim to include 1850 patients. The trial is approved by the Swedish Ethical Review Authority. Results of primary and secondary clinical outcomes and the environmental outcome will be submitted for publication in a peer‐reviewed journal.

**Trial Registration:** NCT06140147.

## 1 Introduction

Sepsis is characterized by life‐threatening organ dysfunction triggered by the body's response to infection [1]. It is estimated that there are 48 million cases of sepsis worldwide annually and 11 million sepsis‐related deaths each year, with the majority in low‐income countries [2]. Septic shock, a subset of sepsis marked by severe circulatory and metabolic irregularities, has a 90‐day mortality of about 45% [3, 4, 5].

Administration of fluids is an essential part of caring for patients with septic shock [6]. Resuscitation fluids are administered to correct hypovolemia, while fluids administered for other indications may collectively be referred to as non‐resuscitation fluids. The latter includes fluids given as vehicles to deliver medication and nutrients, correct electrolyte imbalances, and ensure sufficient hydration (maintenance fluids). While appropriate fluid therapy can undoubtedly save lives, observational studies suggest that large volumes of fluid may have deleterious effects [7, 8, 9, 10], prompting trials to investigate if reducing fluid administration to septic shock patients can improve outcomes.

### 1.1 Previous Trials

A recent systematic review and meta‐analysis of sepsis trials identified 13 trials comparing a restrictive fluid administration to usual care, i.e., more liberal administration [11]. Only one of these trials evaluated an intervention aimed at reducing both resuscitation and non‐resuscitation fluid administration [12], whereas the rest targeted only resuscitation fluids. The meta‐analysis including all 13 trials showed no difference in mortality (relative risk [RR]: 0.97 [97% confidence interval (CI); 0.86–1.09]), possibly due to small differences in fluid volume between the intervention and control groups.

### 1.2 Rationale for a New Trial

We recently conducted a feasibility trial, in which we found that protocolized reduction of non‐resuscitation fluids in septic shock patients resulted in a median reduction of 3.6 L in the total volume of administered fluids within the first 3 days after inclusion, compared with usual care [13]. This reduction is nearly twice as large as the most effective fluid restriction protocol targeting resuscitation fluids [5] and could potentially impact outcomes. In addition, it is unknown whether the balance between the benefits and harms of resuscitation fluids differs from that of non‐resuscitation fluids. Taken together, a trial powered to detect if restrictive administration of non‐resuscitation fluids influences patient‐important outcomes is warranted.

### 1.3 Objective

The objective of this randomized multi‐centre trial is to assess the beneficial and harmful effects of restrictive administration of non‐resuscitation fluids in adult patients with septic shock and to test the hypothesis that reducing the administration of non‐resuscitation fluids improves outcomes.

## 2 Methods and Analysis

### 2.1 Study Setting

This is an investigator‐initiated, multicentre, non‐commercial, parallel‐group, randomized clinical trial including critically ill intensive care unit (ICU) patients at both university and non‐university hospitals. An updated list of study sites can be found on the trial website (reduse‐trial.com) and ClinicalTrials.gov (NCT06140147, registered before enrolment of the first participant; 2023‐11‐14). The full protocol can be found in the Supporting Information and on the trial website. An independent Data Safety Monitoring Committee (DSMC) will monitor the trial according to a DSMC charter.

### 2.2 Eligibility

#### 2.2.1 Inclusion Criteria

- Adult (≥ 18 years of age)
- Septic shock according to the Sepsis 3 [1] criteria at any time within 12 h of ICU admission (suspected or confirmed infection, plasma lactate above 2 mmol/L, and use of vasopressor to maintain mean arterial pressure of 65 mmHg or above after receiving adequate fluid resuscitation [> 1 L within 12 h of screening]) and ongoing vasopressor therapy at the time of screening.

#### 2.2.2 Exclusion Criteria

- Confirmed or suspected pregnancy
- Previous inclusion in the REDUSE trial
- Screened more than 12 h after ICU admission.

Patients readmitted to the ICU during the same hospital stay will remain in the same study arm to which they were initially allocated, regardless of diagnosis. Patients readmitted to the ICU after discharge from the hospital will not be eligible for re‐inclusion.

### 2.3 Intervention

Patients will receive non‐resuscitation fluids according to the allocated treatment within 2 h of randomization. Fluids other than colloids, blood products, or crystalloids administered to correct hemodynamic impairment (as noted in the patients' medical records) will be considered non‐resuscitation fluids. The types of maintenance fluids will be given according to usual care as defined at each centre, with the aim of using similar types of fluids in both groups. The allocated treatment will be continued while the participant is admitted to an ICU participating in the REDUSE trial up to a maximum of 90 days.

#### 2.3.1 The Intervention Group

- Maintenance fluids will be discontinued in participants with a positive cumulative fluid balance who are judged not to be dehydrated by the treating physician.
- Glucose may be used at a maximal dose of 1 g/kg/day using 20% or more concentrated glucose solutions starting at 72 h after inclusion as nutrition if enteral feeding is not tolerated. Glucose at this concentration or higher may be started earlier in patients at risk of hypoglycaemia (blood glucose < 5 mmol/L and trending downwards) and in patients with insulin‐dependent diabetes if enteral feeding is not tolerated and if mandated by local protocol.
- Intravenous medications will be concentrated according to a trial‐specific protocol (Supporting Information).
- Enteral nutrition with an energy density of 2 kcal/mL will be administered according to local protocols.
- Participants with neutral or negative cumulative fluid balance will receive maintenance and other fluids to cover their daily water needs (about 1 mL/kg/h).
- Intravenous fluid and enteral water will be used to correct electrolyte disturbances as needed.
- Parenteral nutrition will be given according to local protocols.

#### 2.3.2 The Usual Care Group

- Non‐resuscitation fluids will be administered according to local routines.
- Unless local protocols dictate otherwise, maintenance fluids (enteral water, crystalloids and/or glucose) will be given at 1 mL/kg/h.
- Unless local protocols dictate otherwise, a maximum glucose concentration of 10% will be used.
- Medications will be concentrated according to local protocols.

Prior to the start of the trial, site investigators will establish what constitutes usual care in the ICU at their site and ensure protocol adherence in the two groups through continuous treatment monitoring.

Resuscitation fluids (albumin, blood products, or crystalloids) will be administered according to local protocols. Crystalloids will be defined as resuscitation fluids if given to correct hemodynamic impairment as noted in the patient chart or at a rate of ≥ 5 mL/kg/h if the indication is unclear [14]. All other care will be according to local routines and not protocolized.

### 2.4 Outcomes

#### 2.4.1 Primary Outcome

- All‐cause mortality at 90 days from inclusion.

#### 2.4.2 Secondary Outcomes

- One or more complications in the ICU, defined as one or more of the following events in the ICU:
- Acute cerebral infarction (documented on brain magnetic resonance imaging or computed tomography scans) with corresponding neurological symptoms.
- Acute coronary syndrome (defined as acute myocardial infarction or unstable angina pectoris) AND either reperfusion treatment (percutaneous coronary intervention [PCI] or thrombolysis) or initiated/increased antithrombotic treatment.
- Acute intestinal infarction (either diagnosed during surgery or by angiography).
- Limb ischemia (defined as clinical signs of limb ischemia and one of the following treatments: open or percutaneous vascular intervention, amputation, or initiation/increased antithrombotic treatment).
- New onset severe acute kidney injury (defined as stage 3 according to the kidney disease improving global outcomes [KDIGO] criteria [15])
- Mechanical ventilation‐free days within 90 days of inclusion.
- Cognitive function as determined by the Montreal Cognitive Assessment 7.1 BLIND/telephone version (MoCA‐BLIND) at 6 months after inclusion [16, 17].
- Health‐Related Quality of Life as determined by the EQ Visual Analogue Scale (EQ‐VAS) at 6 months from inclusion [18].

#### 2.4.3 Primary Environmental Outcome

- Climate impact within 90 days after inclusion modeled as the Global Warming Potential (GWP) of emitted greenhouse gases over a 100‐year period using a life cycle assessment methodology. GWP is defined as radiative forcing based on the change in concentration of greenhouse gas emissions, taking into account the residence time of the substances and is expressed as carbon dioxide equivalents (CO_2_eq) [19].

#### 2.4.4 Exploratory Outcomes

- Hospital‐free days within 90 days of inclusion.
- Vasopressor‐free days within 90 days of inclusion.
- Renal replacement therapy (RRT)‐free days within 90 days of inclusion.
- Major adverse kidney events (MAKE) at 90 days, defined as the composite of mortality at 90 days from inclusion or initiation of new renal replacement therapy or persistent renal dysfunction (defined as a final inpatient creatinine level ≥ 200% of the baseline value) within 90 days of inclusion.
- Cumulative dose of diuretics during the first 5 days of inclusion, as measured in defined daily doses per the World Health Organization (WHO) [20].
- Overall functional outcome by Glasgow Outcome Scale Extended (GOSE), at 6 months after inclusion [21].
- EQ‐5D‐5 levels level scores and sum of level scores (EQ‐5D‐5L) at 6 months after inclusion.
- WHO Disability Assessment Schedule (WHODAS) 2.0 (12‐item version), at 6 months after inclusion [22].
- Modified Fatigue Impact Scale (MFIS) at 6 months after inclusion [23].
- All‐cause mortality at 12 months after inclusion.
- Number of days in the ICU within 90 days of inclusion.
- Hypoglycaemia (moderate 3.9–2.3 mmol/L and severe ≤ 2.2 mmol/L) in the ICU.
- Hypernatremia (> 159 mmol/L) in the ICU.
- Acid–base disturbances, defined as hyperchloremic acidosis (pH < 7.15 and plasma Cl‐ > 115) or metabolic alkalosis (pH > 7.59 and S‐BE > 9) in the ICU.
- Central venous catheter‐related complications potentially linked to concentrated drugs administered in the intervention group (e.g., thrombosis, stenosis, malfunction, and infections) in the ICU.

#### 2.4.5 Suspected Unexpected Serious Adverse Events

In addition, we will monitor and report:

- Suspected unexpected serious adverse events (SUSAE) (an adverse event not reasonably explained by other factors than the intervention, which may cause death or be life‐threatening, prolong hospitalization, or may result in significant disability/incapacity).

All SUSAEs and their circumstances will be recorded in the electronic case report form (eCRF). The site investigator will assess the causality between the trial intervention and the SUSAE and will follow participants until symptoms of the event are resolved. A qualified physician in the trial management team will assess each SUSAE report for safety. The DSMC will monitor and assess all SUSAEs at the interim analysis.

#### 2.4.6 Secondary Environmental Outcomes

In addition to the climate impacts, other environmental impacts of the intervention will be estimated using life cycle assessment methodology [24]. A separate detailed protocol outlining the boundaries of the climate‐ and environmental analysis, the impact assessment methodology, and the statistical analysis plan will be submitted for publication before the last patient is randomized.

### 2.5 Procedures for Screening and Recruitment

All patients admitted to participating ICUs with a diagnosis of septic shock within 12 h of ICU admission will be screened. Information sessions will be held to ensure that key medical personnel at all sites are informed of the trial. Reasons for not including eligible patients will be documented. Informed consent will be obtained according to the respective national ethical approval.

### 2.6 Assignment of Interventions

An internet‐based randomization module integrated into the eCRF (Spiral Software, Wellington, New Zealand) will randomly assign patients to receive either protocolized restrictive administration of non‐resuscitation fluids or usual care in a one‐to‐one ratio. This will enable the proper generation and concealment of the allocation sequence until the intervention is assigned. The randomization process will be stratified for each trial site using permuted blocks of varying sizes unknown to the trial investigators.

### 2.7 Participant Timeline

**FIGURE 1:**
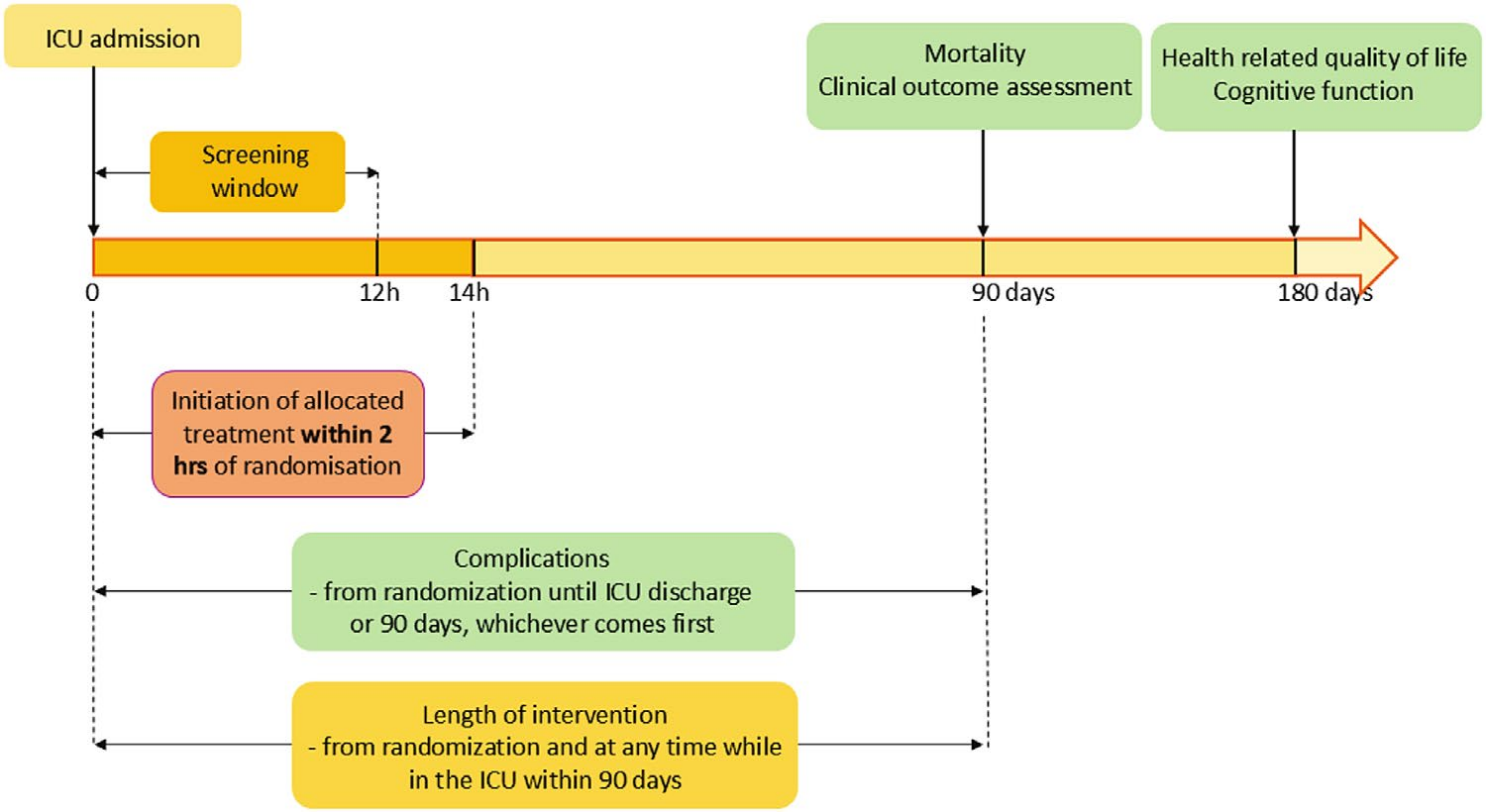
Trial timeline. Vertical arrows indicate specific time points for events or assessments, whereas horizontal arrows indicate time periods. Complications: Cerebral, cardiac intestinal or limb ischaemia or new onset grade 3 acute kidney injury.

Participants will receive non‐resuscitation fluids according to the assigned intervention within 2 h of inclusion, and the intervention will be applied whenever the participant is admitted to a REDUSE ICU for a maximum of 90 days after inclusion. A blinded outcome assessor will follow up on surviving participants at 6 months after inclusion and record their 1‐year survival status (please see Figure 1 for the timeline).

### 2.8 Blinding

The participants and their relatives will not be informed about group allocation. The steering group, trial investigators, assessors of patient‐centered outcomes at 6 months, trial statisticians, central data monitoring committee, manuscript authors, and the DSMC will be blinded to group allocation. Due to the complexity of the intervention, the healthcare providers who care for the participants at their bedside and the research staff collecting data up to 90 days will not be blinded. The two groups will be coded as “A” and “B” during data analysis and preparation of the main manuscript. The author group must approve all conclusions regarding the outcome of intervention groups “A” and “B” before the randomization code is broken. The DSMC may request unblinding if necessary.

### 2.9 Data Collection

Clinical, laboratory, and background data will be collected at enrolment, during ICU stay, at ICU discharge, at 90 days after inclusion, and at the 6‐month and 12‐month assessments. Trained site personnel will gather data from hospital records and, if needed, from relatives/close friends of the participant and enter it into a web‐based case report form (eCRF). The trial coordinating team will transfer data from the eCRF to a trial database. Site investigators will verify the validity and completeness of records. A detailed description of the data to be collected is provided in the Supporting Information.

A study‐trained and blinded outcome assessor will perform the MoCA‐BLIND and GOSE during a structured telephone interview at 6 months after inclusion. Before the follow‐up, the patient‐reported questionnaires EQ‐5D‐5L, WHODAS, and MFIS will be sent to the participants to complete and return via a pre‐paid envelope, or alternatively, completed during the follow‐up. To promote participant retention, other modes of follow‐up, such as home or audio‐visual web‐based meetings, can be used. If the trial participant cannot complete the patient‐reported outcome measures, e.g., due to poor health or cognitive impairment, proxy rating, by a close relative or friend, may be used to minimize missing data. An authorized interpreter will be used if needed.

### 2.10 Data Management

Data will be entered into patient‐specific trial ledgers or directly into the eCRF. Site principal investigators will be responsible for training the research staff in proper data handling. Predefined values and ranges on all data entries will be built into the eCRF to prevent data entry errors and promote data quality. Central data monitoring will ensure the quality and completeness of the collected data, follow‐up rates, and adherence to the protocol during the study [25]. A detailed central data monitoring plan will be presented in a separate publication.

Individual patient data will be treated as ordinary chart records and kept according to each participating country's legislation (e.g., data protection agencies). Information will be entered into a web‐based eCRF that meets the criteria for handling patient data as outlined in national legislation on personal data management in participating countries and the General Data Protection Regulation of the EU (European Parliament and Council of the European Union directive 2001/20/EC) and the guidelines for electronic signatures set by the Federal Drug Administration (FDA 21 CFR Part 11 Guidelines for Electronic Signatures). The original records and trial database will be preserved at trial sites or at the trial administration sites for 15 years to facilitate inspection by relevant authorities. The trial database will be anonymized for requested revisions.

### 2.11 Sample Size and Power Calculations

#### 2.11.1 Sample Size Calculation for Primary Outcome

The sample size calculation is based on an expected 90‐day mortality of 45% in the control group [3, 4, 5, 26]. To detect an absolute reduction in mortality of 7.5%, with an alpha of 5% and a power of 90%, the required sample size is 1808. We estimated an intervention effect of 7.5% because it has been observed in previous large intensive care trials assessing interventions in critically ill patients [26, 27], and it is a clinically relevant effect size. We will include a total of 1850 patients, accounting for loss to follow‐up and withdrawal of consent.

#### 2.11.2 Power Calculations for Secondary Outcomes

Based on a relative risk reduction of 20% and a rate of complications in the control group of 30%, the sample size yields a power of 99% for the complications outcome [5].

Based on a difference of 2 mechanical ventilation‐free days, and the data collected in the REDUSE feasibility trial [13], we used simulations to ascertain the power using the van Elteren test as the primary analysis. Using 1000 iterations, we found that the sample size yields a power of 80% for the mechanical ventilation‐free days outcome.

Based on a minimal important difference of 1.5, a standard deviation of 2.8 [28], the sample size yields a power of 100% for the MOCA‐BLIND score.

Based on a minimal important difference of 5 points, a standard deviation of 20 points, the sample size yields a power of 99% for the EQ‐VAS score [29, 30, 31].

### 2.12 Statistical Methods

A detailed statistical analysis plan will be published before the randomization of the last participant. In short, two independent and blinded statisticians will analyse data on an intention‐to‐treat basis. The intention‐to‐treat population comprises all randomized patients who consented to the use of their data. The per‐protocol population consists of all randomized patients who consented to the use of their data, excluding those with one or more specified protocol deviations (defined as randomization of a non‐eligible patient and non‐compliance with the treatment algorithm). All analyses will be adjusted for site of admission. The primary outcome and the first secondary outcome (complications) will be analysed using mixed effects logistic regression with site as a random intercept. Relative risks will be estimated using the “nlcom” STATA command or G‐computation in R (R Core Team, Vienna, Austria). Count data (mechanical ventilation‐free days) will be analysed by van Elteren test stratified by site. Secondary continuous outcomes (MoCA‐BLIND and EQ‐VAS) will be analysed using mixed effects linear regression with site as a random intercept.

All primary conclusions will be based on our primary outcome, and secondary and explorative outcome results will be considered as hypothesis generating. Hence, a *p*‐value less than 0.05 will be the threshold for statistical significance for all analyses.

#### 2.12.1 Missing Data

We estimate that less than 5% of values will be missing for the primary and secondary outcomes except for cognitive function and HRQoL. For the latter outcomes, we expect less than 15% of missing data. Missing data will be managed as described in detail previously [32, 33]. In short, based on assessments of missing data patterns, we will in secondary analyses consider using multiple imputation and present best‐worst and worst best case scenarios. Best‐worst and worst‐best case scenarios assess the potential range of impact of the missing data for the trial results. In the “best‐worst” case scenario, it is assumed that all patients lost to follow‐up in the intervention group have had a beneficial outcome (have survived etc.), and all those with missing outcomes in the control group have had a harmful outcome (have not survived etc.) and so forth. Conversely, in the “worst‐ best” case scenario, it is assumed that all patients who were lost to follow‐up in the experimental group have had a harmful outcome and that all those lost to follow‐up in the control group have had a beneficial outcome. When continuous outcomes are used, a “beneficial outcome” will be defined as the group mean plus two SDs of the group mean (fixed imputation), and a “harmful outcome” will be defined as the group mean minus two SDs of the group mean (fixed imputation).

#### 2.12.2 Subgroup Analyses

The heterogeneity of the intervention effect on the primary and secondary outcomes will be assessed in the following subgroups based on baseline characteristics at inclusion.

- Mechanical ventilatory support at the time of randomization (defined as invasive or non‐invasive mechanical ventilation or nasal high flow treatment) (yes/no)
- Acute kidney injury at the time of randomization (KDIGO stage ≥ 1 [yes/no])
- Sex (male/female)
- Age (≥ 65 years [yes/no])
- Frailty by Clinical Frailty Scale (CFS ≤ 3, 4, and > 5).
- Weight at admission (above/below the median of the intention to treat population)
- Validated infection (Linder‐Mellhammar score ≥ 3 [yes/no]) [34])
- Intravenous fluid at time of randomization < 30 mL/kg (yes/no)
- Plasma Lactate > 4 mmol/L (yes/no)

### 2.13 Biobank

We will collect blood samples at inclusion and at the start of days 3 and 5 after inclusion. All samples will be stored centrally once the trial is completed. Blood samples will be processed, aliquoted, and analyzed according to separate protocols. Biosample analyses will be described in separate manuscripts, and no analysis will occur before the trial ends. No results from the biosamples will be published in the initial manuscript. Participation in the collection of biosamples will be optional for each site.

### 2.14 Ethics and Informed Consent

Ethics applications will be submitted to National Research Ethics boards in participating countries. The applications will seek approval for a deferred consent process. This is based on the premise that, to have the greatest possible impact, the intervention must be started as soon as possible after admission to the ICU. Because cognitive impairment is a hallmark symptom of septic shock, we regard it as impossible in most cases to obtain informed consent at the time of presentation [1, 35]. We judge that this strategy is justifiable according to the Declaration of Helsinki Article 30 available from the World Medical Association [36]. Surviving participants will be asked for written consent to continue participating in the trial as soon as they can make an informed decision. Please see the Supporting Information for a sample consent form.

Participants are free to withdraw their consent at any time. If consent is withdrawn or not given, participants will be asked if the data acquired up to that time point can be used and included in the analyses, and if the data for the primary endpoint can be collected. If the participant declines, all data, except initials, sex, and dates of screening and withdrawal, will be destroyed.

The first version of the protocol was approved by the Swedish Ethical Review Authority on 8 February 2021 (#2020‐06594). The study was registered on clinicaltrials.gov (NCT06140147) on 18 November 2023.

### 2.15 Patient and Public Involvement

A patient organization for sepsis patients (Sepsisföreningen) has reviewed the protocol and endorsed the trial objectives. Based on feedback from the patient organization, we have included the WHODAS 2.0 and the MFIS questionnaires in our follow‐up. The patient organization will be consulted concerning any changes in the protocol deemed important from a participant's perspective (i.e., ethical implications of protocol changes).

### 2.16 Trial Conduct

This trial will be carried out in adherence to good clinical research practices and in accordance with the most recent edition of the Declaration of Helsinki [36].

### 2.17 Data Safety Monitoring

An independent DSMC will monitor the trial according to the charter for the DSMC (please see Supporting Information). The DSMC will collaborate with an independent statistician to conduct a blinded interim analysis, reviewing data related to treatment efficacy (primary outcome), safety, and the quality of trial conduct, after obtaining 90‐day follow‐up data for the first 400 patients. Lan‐DeMets group sequential monitoring boundaries will determine if the trial should be terminated [37]. If necessary, the DSMC will be able to request the data to be unblinded. The DSMC may also request additional analyses based on the results of the planned interim analysis.

### 2.18 Monitoring

The trial will be monitored by national monitoring offices in participating countries. All sites will participate in a site initiation meeting with an external monitor before the start of inclusion to ensure that the study can be performed according to protocol and that the essential study documents are prepared. One monitoring visit will be done at each site when 10 subjects have been randomized at the site. If important deviations have occurred, an extra monitoring visit to the site may be scheduled in agreement with the principal investigator. Monitors will also conduct a close‐out visit at all sites, including control of routines for data collection, data entry, and source data verification for a selected subset of the data.

## 3 Discussion

### 3.1 Strengths

The strengths of this trial include the generalizability inherent in a pragmatic multicenter trial, where both university and non‐university hospitals will recruit patients with few exclusion criteria. Another strength is that the effectiveness of the intervention in reducing fluid volume has been demonstrated in our feasibility trial [13]. In addition, the feasibility trial allowed us to identify opportunities to fine‐tune the protocol and the eCRF. For example, to further reduce the fraction of missing data at the 6‐month follow‐up, we have implemented regular central data monitoring to identify sites that need additional training and support.

### 3.2 Limitations

A limitation of this trial is the increased awareness of the risks of fluid overload in the critical care community. This may lead to a drift in practice in the usual care group toward a more restrictive prescription of non‐resuscitation fluids. This risk is compounded by the fact that strategies for administering non‐resuscitation fluids are not mentioned in guidelines for the care of patients with septic shock, and some sites may not have written protocols for administering maintenance fluids and glucose [38]. Several measures were implemented to address this concern in our recent feasibility trial, such as encouraging site investigators, at sites that did not have written protocols for administration of non‐resuscitation fluids, to establish written protocols reflecting local practice. A similar approach will also be implemented in this trial. The separation between the groups in our feasibility trial indicated these measures were successful. To further mitigate the risk of drift, we will use central data monitoring for trimonthly assessments of separation between the groups. This will allow for the timely identification of drift in the usual care group, which can be communicated to site investigators. Another potential limitation is that the research staff responsible for data collection up to 90 days post‐inclusion will not be blinded.

## 4 Dissemination

The main trial findings, including the primary and secondary clinical outcomes and the primary environmental outcome, will be submitted to a peer‐reviewed international journal. Exploratory outcomes and biosample studies will be reported in separate manuscripts. The data from the trial will be owned by the sponsor and will be administered by the sponsor in conjunction with the Steering Group of the Trial (as defined in the Trial protocol). After the main publication, the site investigators will receive access to site‐specific data to be used at their discretion.

## 5 Data Sharing

Approximately 1 year after publication of the main report, individual de‐identified data will be available for non‐REDUSE researchers who provide a methodologically sound proposal as judged by the steering committee. A data access agreement will need to be signed to gain access.

## 6 Study Status

Recruitment started on November 27, 2023, and is predicted to end in 2027.

## 7 Protocol and Amendments

The protocol version outlined herein is V.1.4. Protocol modifications will be promptly updated on ClinicalTrials.gov, the trial website, and communicated to all site investigators. Modifications to the protocol will be subjected to ethical review as required.

## Author Contributions

P. Bentzer drafted the manuscript. All other authors contributed to the study design and critically revised the manuscript. All authors approved the final version.

## Conflicts of Interest

The authors declare no conflicts of interest.

## Supporting information


**Data S1.** Supporting Information.

## Data Availability

Data sharing is not applicable to this article as no new data were created or analyzed in this study.
