# Supplementary material for: Protocolized REDUction of Non‐Resuscitation Fluids in SEptic Shock Patients. A Protocol for the REDUSE Randomized Clinical Trial
Source: Acta Anaesthesiol Scand. 2025 Jul 16;69(7):e70095. doi: 10.1111/aas.70095 (PMC12267905; doi:10.1111/aas.70095)
Supplement: Supplementary file 1 — Data S1. Supporting Information. [file AAS-69-0-s001.pdf]

Clinical Trial Protocol

Protocolized REDUction of non-resuscitation fluids versus usual care in SEptic shock patients. A protocol for the REDUSE randomised clinical trial

Version 1.4

Contents

1. Trial overview 4
2. Background and study rationale 4
2.1 Background 4
2.3 Previous evidence 4
2.4 Rationale for a new trial 5
3. Trial hypothesis and outcomes 5
3.1 Primary outcome 5
3.2 Secondary outcomes 5
3.2 Primary environmental outcome 6
3.3 Explorative outcomes 6
3.4 Rationale for primary and secondary outcomes 6
4. Eligibility 7
4.1 Inclusion criteria 7
4.2 Exclusion Criteria 7
4.3. Note on inclusion and exclusion criteria 7
4.4 Exit from the trial 8
5. Trial design 8
5.1 Screening and randomisation 8
5.2 Intervention 8
5.2.1 The intervention group 9
5.2.2 Usual Care group 9
5.3 Follow up 9
5.4 Blinding 10
5.5 Definitions 10
5.5.1. Days 10
5.5.2. Fluid balance 10
5.5.3. Protocol deviations 10
5.6 Co-enrolment 10
6. Data collection 10
6.1 Quality control of data 11
6.2 Biobank 11
7. Ethics and Informed consent 11
8. Data management 12
8.1 Data handling and record keeping 12
8.2 Quality control and quality assurance 12
9 Adverse events 12
9.1 Definitions 12
9.2 Reporting of SUSAEs 13
10. Statistical analysis plan 13
10.1 Sample size 13

10.1.1 Primary outcome 13
10.1.2 Secondary outcomes 13
10.2 Analysis methods 14
10.3 Missing data 14
10.4 Subgroup analysis 14
10.4 Exploratory analyses 15
10.5 Statisticians 15
10.6 Data safety monitoring committee 15
10.7 Members of the data safety monitoring committee 15
10.8 Environmental analysis 15
10.9 Health economic analysis 16
11. Publication of Data 16
11.1 Data sharing 16
13. Funding 16
14. Timeline 16
14.1 One year following the start of the project (end of 2024). 17
14.2 Two years following the start of the project (end of 2025). 17
14.3 Three years following the start of the project (end of 2026 17
14.4 Four years following the start of the project (end of 2027). 17
15. Investigators 17
15.1 Management group 17
15.2 Steering group 17
References 18
Table 1. Study Schedule 21
Appendix A. Description of collected data 22
Appendix B. Charter for the Data Safety and Monitoring Committee (DSMC) 29
1. Introduction 29
2. Primary responsibilities of the DSMC 29
3. Members of the DSMC 29
3.1 Members of the DSMC 30
4 Conflicts of interest 30
5. Meetings of the DSMC 30
6. Communication between the steering group and the DSMC 30
7. Minutes of the DSMC meetings 30
8. Recommendations to the steering group 31
9. Statistical monitoring guidelines 31
10. Conditions for transfer of data from the steering group to the DSMC 32
Appendix C. Treatment algorithm for non-resuscitation fluids in the intervention arm. 33
Appendix D. Medications in the intervention group. 34
Appendix E. Model patient information and informed consent form 41

1. Trial overview

The REDUSE trial is a multicentre, investigator-initiated, randomized clinical superiority trial comparing protocolized restrictive strategy for the administration of non-resuscitation fluids with usual care in participants with septic shock. Adult patients with septic shock will be eligible for inclusion. Patients will be randomized within 12 hours of admission to the intensive care unit. Participants will not receive maintenance fluids in the intervention arm unless the total fluid volume is insufficient to provide hydration. All intravenous drugs and nutrition will be concentrated to reduce fluid volume. Resuscitation fluids will be administered according to local routines. The intervention will last for the duration of the intensive care unit stay. Participants in the control arm will receive usual care. The primary outcome will be all-cause mortality at 90 days. Secondary outcomes will be complications during ICU stay, cognitive function, health-related quality of life at 6 months, and days alive without mechanical ventilation at 90 days. Healthcare staff involved in the participants' immediate care will not be blinded to the intervention, but participants, outcome assessors, statisticians, manuscript authors and the data safety monitoring committee will be blinded to treatment allocation.

2. Background and study rationale

2.1 Background

Sepsis is defined as life-threatening organ dysfunction caused by a host response to infection (Singer 2016). Recent estimates suggest that 48 million cases of sepsis occur globally every year and that 11 million sepsis-related deaths occur annually, with the majority of cases occurring in developing countries (Rudd 2020). Septic shock is a subgroup of sepsis with particularly severe circulatory and metabolic abnormalities and 90-day mortality is about 45% (Hernandez 2019, Holst 2014, Meyhoff 2022).

Administration of fluids is an essential component of caring for patients suffering from septic shock (Evans 2021). Fluids are administered for different reasons. Resuscitation fluids are administered intravenously to correct hemodynamic impairment. In contrast, non-resuscitation fluids are administered intravenously and enterally as vehicles for medications and nutrition, to correct electrolyte disturbances, and to ensure adequate hydration (maintenance fluids). The latter purpose is considered to require a total of about 1-2 litres of fluids per day (1 ml/kg/h) in healthy humans and may increase in pathophysiological conditions due to higher than normal losses. More than 50% of patients with septic shock receive 4 L or more of fluids on the first day in the ICU (Marik 2017). This may be adequate in patients with pre-existing deficits, but data suggest that large volumes of fluids are not without risks. Non-randomised studies have indicated that excessive fluid administration might have detrimental adverse effects such as tissue oedema with impaired oxygen delivery and organ function, compartment syndromes and ultimately increased mortality (Boyd 2011, Marik 2017, Silversides 2018, Sakr 2017). This has formed the basis for trials comparing restrictive administration of resuscitation fluids with usual care in adult septic shock patients.

2.3 Previous evidence

A recent systematic review with meta-analysis identified 13 trials comparing a restrictive approach of fluid administration with usual care in adult patients with sepsis and/or septic shock (Sivapalan 2023). Twelve of these trials assessed interventions with the objective to

reduce administration of only resuscitation fluids. One trial assessed interventions to reduce resuscitation and non-resuscitation fluids (Chen 2015). A meta-analysis including all 13 trials, showed no difference in mortality (relative risk [RR]: 0.97 [97% CI; 0.86-1.09]), possibly due to small differences in fluid volume between the intervention and control groups.

2.4 Rationale for a new trial

Based on data showing that non-resuscitation fluids are the major source of fluid after the first day in the ICU we recently performed a feasibility trial comparing a protocolized reduction of non-resuscitation fluids to usual care in septic shock (Lindén-Søndersø 2019, Lindén 2023, Linden 2024). Our data shows that this approach may reduce the total volume of administered fluids by a median of 3.6 (95% CI; 1.6 to 5.3) L in the first 3 days after inclusion. This reduction is almost twice as large as the most effective protocol targeting the restriction of resuscitation fluids (Meyhoff 2022) and could potentially impact outcomes. In addition, it is unknown whether the balance between benefits and harms of resuscitation fluids differs from that of non-resuscitation fluids. Taken together, a trial powered to detect if restrictive administration of non-resuscitation fluids influences patient-important outcomes is warranted.

3. Trial hypothesis and outcomes

The objective is to assess the beneficial and harmful effects of a restrictive strategy for administration of non-resuscitation fluids in adult patients with septic shock.

3.1 Primary outcome

- All-cause mortality at 90 days

3.2 Secondary outcomes

- One or more complications in the ICU defined as one or more of the following events in the ICU:
    a) Acute cerebral infarction (documented on brain MRI or CT scans) with corresponding neurological symptoms.
    b) Acute coronary syndrome (defined as acute myocardial infarction or unstable angina pectoris) AND either reperfusion treatment (percutaneous coronary intervention, PCI or thrombolysis) or initiated/increased antithrombotic treatment.
    c) Acute intestinal infarction (either diagnosed during surgery or by angiography).
    d) Limb ischemia (defined as clinical signs of limb ischemia AND one of the following treatments: open or percutaneous vascular intervention, amputation, or initiation/increased antithrombotic treatment.
    e) New onset severe acute kidney injury (defined as stage 3 according to the kidney disease improving global outcomes [KDIGO] criteria [Kellum 2013]).
- Mechanical ventilation-free days within 90 days of inclusion.
- Cognitive function as determined by the Montreal Cognitive Assessment 7.1 BLIND/telephone version (MoCA-BLIND) at 6 months (Pendlebury 2013, Wittich 2010).

- Health-Related Quality of Life as determined by the EQ Visual Analogue Scale (EQ-VAS) at 6 months (Van Reenen 2015).

3.2 Primary environmental outcome

- Climate impact within 90 days after inclusion modelled as Global Warming Potential (GWP) over a 100-year period using a life cycle assessment methodology. GWP is defined as radiative forcing based on the change in concentration of greenhouse gas emissions taking into account the residence time of the substances and is expressed as carbon dioxide equivalents (CO2eq) (Dhalcal 2022).

3.3 Explorative outcomes

- Hospital-free days within 90 days of inclusion
- Vasopressor-free days within 90 days of inclusion.
- Renal replacement therapy (RRT)-free days within 90-days of inclusion.
- Major adverse kidney event (MAKE) at 90-days, defined as the composite of mortality at 90 days, initiation of new of renal replacement therapy, or persistent renal dysfunction (defined as a final inpatient creatinine value ≥200% of the baseline value within 90 days)
- Cumulative dose of diuretics during the first 5 days of inclusion, as measured in defined daily doses per the World Health Organization (WHO).
- Glasgow Outcome Scale Extended (GOSE) at 6 months (McMillan 2016).
- European Quality of Life visual 5 dimension-5 level scale (EQ-5D-5L) at 6 months.
- WHO Disability Assessment Schedule (WHODAS) 2.0 (12 item version) total score at 6 months (Üstün 2010).
- Modified Fatigue Impact Scale (MFIS) at 6 months (Téllez 2005).
- All-cause mortality at 12 months.
- Number of days in the ICU within 90 days of inclusion
- Hypoglycaemia (moderate 3.9 - 2.3 mmol/l and severe ≤ 2.2 mmol/l) in the ICU
- Hypernatremia (> 159 mmol/L) in the ICU
- Acid base disturbances (hyperchloremic acidosis [pH < 7.15 and plasma Cl^- > 115], metabolic alkalosis [pH > 7.59 and S-BE > 9]) in the ICU
- Central venous catheter related complications that could potentially be related to concentrated drugs given in the intervention group (for example, thrombosis, stenosis, malfunction, and infections) in the ICU

For Swedish sites mortality data will be collected from the National board of Health and Welfare.

3.4 Rationale for primary and secondary outcomes

Several observational studies have reported an association between a positive cumulative fluid balance and mortality (Boyd 2011, Marik 2017, Sakr 2017, Silversides 2018).

Because less administration of non-resuscitation fluids could decrease intravascular volume and therefore increase ischemia and acute kidney injury, we will assess the incidence of ischemic complications and acute kidney injury. Previous trials assessing effects of restrictive

administration of resuscitation fluids in sepsis have found no effect or a decreased incidence of acute kidney injury (Douglas 2020, Hjortrup 2016, Meyhoff 2022)

Days alive and free of mechanical ventilation is a patient-important outcome and is also essential to assess potential effects on healthcare costs. Strategies to reduce fluid overload in patients suffering from acute respiratory distress syndrome have been suggested to increase the number of days alive and free of mechanical ventilation (Wiederman 2006).

Cognitive impairment and decreased HRQoL are common after sepsis (Hammond 2020, Iwashyna 2013, Kjær 2023). Restrictive fluid administration has been associated with impaired cognitive function in patients suffering from acute respiratory distress syndrome (ARDS) (Mikkelsen 2012). No differences in HRQoL and cognitive function could be demonstrated by restricting resuscitation fluids in septic shock in the recent CLASSIC trial but clinically important differences could not be excluded (Kjær 2023). Based on a modified Delphi process involving patients and researchers (http://www.improvelto.com), the performance-based cognitive screening measure MoCA and the patient-reported outcome measure EQ-5D-5L have been the recommended tests for cognitive function and HRQoL in patients with acute respiratory failure, a common organ failure in septic shock (Needham et al 2017). These tests have been used in large trials in septic shock and have been translated into many languages including Swedish (Kjær 2023, Hammond 2020).

4. Eligibility

Patients will be eligible for inclusion if they fulfil all of the inclusion criteria and none of the exclusion criteria.

4.1 Inclusion criteria

- Adult (≥ 18 years of age)
- Septic shock according to the Sepsis 3 criteria (Singer-16) at any time within 12 hours after ICU admission (suspected or confirmed infection, plasma lactate above 2 mmol/L, and use of vasopressor to maintain mean arterial pressure of 65mmHg or above after receiving adequate fluid resuscitation [> 1L within 12 h of screening]) and ongoing vasopressor therapy at the time of screening.

4.2 Exclusion Criteria

- Confirmed or suspected pregnancy
- Previous inclusion in the REDUSE-trial
- Screened more than 12 hours after ICU admission.

4.3. Note on inclusion and exclusion criteria

Patients with septic shock receive large amounts of fluids and they are the patient category in which an association between a positive fluid balance and a poor outcome has been consistently observed in observational studies. Patients with septic shock have a high mortality and are the most common group of critically ill patients in which large volumes of fluids are administered. Hence, this is a patient group in which the intervention is expected to have the greatest effect.

Patients readmitted to the ICU during the same hospital stay will remain in the same study arm to which they were initially allocated regardless of diagnosis. Patients readmitted to the ICU after discharge from hospital will not be eligible for inclusion.

4.4 Exit from the trial

A participant or his/her surrogate is free to withdraw from the trial at any time. A participant will exit the trial if the participant withdraws her/his consent. The participant making the withdrawal of consent will be asked for permission to use the data acquired up to that time point and if data for the primary endpoint can be collected. If permission is obtained, the participant will be included in the final analyses. If the participant declines, all data with the exception of initials, sex and date of withdrawal will be destroyed. If the trial intervention is discontinued by the treating physician because of adverse events, if the participant is withdrawn from active care and/or the focus of care is changed to palliation, or any other reason, this does not constitute withdrawal from the trial and the participant will not exit the trial (data collection will continue). A participant transferred to a non-REDUSE ICU will not exit the trial. All participants randomized in this trial will be analysed on an intention-to-treat basis.

5. Trial design

The trial is a multicentre, randomized trial with a 1:1 concealed allocation. Participants will receive either protocolized restrictive administration of non-resuscitation fluids or usual care. The trial will be investigator-initiated and non-commercial. The steering group, author group, trial statistician, outcome assessors, prognosticators and the trial coordinating team will be blinded to group allocation. Please see Table 1 for a study schedule.

5.1 Screening and randomisation

Clinical investigators at each participating ICU will be responsible for screening of all admitted patients with a diagnosis of septic shock. A screening log will be compiled while the trial is active at each site and will include all adult patients with an admission diagnosis of septic shock whether they are eligible for inclusion or not. We will document reasons for not including an eligible patient. Informed consent will be obtained as specified in each national ethical approval. Trial sites will have access to an internet-based randomization application within the eCRF to allow for immediate allocation and adequate generation and concealment of the allocation sequence. Each patient will be assigned a unique trial number. Randomization will be performed with permuted blocks of varying block size unknown to the trial investigators, stratified for trial site.

5.2 Intervention

Participants will receive non-resuscitation fluids according to the protocol described below within two hours of randomization. Non-resuscitation fluids are defined as fluids other than colloids, blood products, or crystalloids administered to correct hemodynamic impairment as noted in the participants' charts. The type of maintenance fluids will be given according to usual care at each respective centre with the objective to use similar types of fluids in both groups. The allocated treatment will be continued while the participant is admitted in an ICU participating in the REDUSE trial up to a maximum of 90 days. Patients readmitted to an ICU

participating in the REDUSE trial within 90 days of inclusion will receive the same treatment to which they were initially allocated regardless of admission diagnosis.

5.2.1 The intervention group

- Maintenance fluids will be discontinued in participants with a positive cumulative fluid balance who are judged not to be dehydrated by the treating physician.
- Glucose may be used at a maximal dose of 1g/kg/day using 20% or more concentrated glucose solutions starting at 72 h after inclusion as nutrition if enteral feeding is not tolerated. Glucose at this concentration or higher may be started earlier in patients at risk of hypoglycaemia (blood glucose <5 mmol/l and trending downwards) and in patients with insulin dependent diabetes if enteral feeding is not tolerated and if mandated by local protocol.
- Intravenous medications will be concentrated according to a trial specific protocol (Appendix D).
- Enteral nutrition will have an energy density of 2 kcal/ml and administered according to local protocols.
- Participants with neutral or negative cumulative fluid balance will receive maintenance fluids and other fluids such that the total dose covers their daily water need (about 1ml/kg/h).
- Intravenous fluid and enteral water will be used to correct electrolyte disturbances as needed.
- Parenteral nutrition will be given according to local protocol.

5.2.2 Usual Care group

- Non-resuscitation fluids will be administered according to local routines.
- Unless the local protocols dictates otherwise, maintenance fluids (enteral water, crystalloids and/or glucose) will be given at 1 ml/kg/h.
- Unless local protocol dictates otherwise, a maximum glucose concentration of 10% will be used.
- Medications will be concentrated according to local protocol.

Site investigators will establish what constitutes usual care in their ICU prior to initiation of the trial. Site investigators will ensure protocol adherence in the two groups through continuous treatment monitoring.

Resuscitation fluids (albumin, blood products, crystalloids given to correct hemodynamic impairment as noted in patient chart or at a rate of ≥ 5ml/kg/h if indication is unclear) will be administered according to local protocol (Finfer 2010). All other care will be according to local routines and not protocolized.

5.3 Follow up

At 6 months, a specially trained and blinded outcome assessor will perform a structured telephone interview and administer the MoCA-BLIND, EQ-5D-5L, GOSE, WHODAS and MFIS

evaluations. In cases were the participant´s outcome is too poor to complete the tests, a relative or close friend will be asked to rate the participant´s HRQoL by the EQ-5D-5L test. The outcome assessor may be an occupational therapist, a physician, a research nurse, or a psychologist. Outcome assessors will receive a trial manual with detailed guidelines for performing the questionnaires and assessments. Training sessions will be provided by the trial coordinating team. To avoid missing data alternative strategies will be used. These include inviting the participant to visit a clinic, visiting the participants' home or an audio-visual web-based meeting. If needed an authorized interpreter will be used.

5.4 Blinding

The clinical team caring for participants bedside and the research staff collection data up to 90 days after inclusion will not be blinded due to the nature of the intervention. The participants and their family will not be informed about group allocation. The steering group, author group, trial statisticians, outcome assessors at 6 months and at one year, the trial coordinating team, manuscript writers and the data safety and monitoring committee will be blinded to group allocation. The two groups will be coded as "A" and "B". Two conclusions from all outcomes in the main manuscript will be drawn: one assuming "A" is the intervention group and "B" is the control group - and one assuming the opposite. All conclusions must be approved by the author group before the code is broken.

5.5 Definitions

5.5.1. Days

Day 1 is from time of randomization to start of new 24 hr period as per local protocol. Day 2 is next 24 hr period. The last day of ICU stay is from the start of new 24 hr period as per local protocol until discharge.

5.5.2. Fluid balance

Fluid balance is calculated as sum of all input of enteral and parenteral fluids minus all measured losses. Estimated loss through evaporation will not be included in fluid balance. Stool will not be included in balance unless the patient has a faecal management system or similar device in place.

5.5.3. Protocol deviations

Protocol deviations include randomization of a non-eligible patient and non-compliance with treatment algorithm in the intervention and control arms as described above.

5.6 Co-enrolment

We will encourage co-enrolment with other interventional trials, unless the trial protocols are incompatible. Co-enrolment agreements will be established with the sponsor/principal investigator of such trials and a list of trials approved for co-enrolment will be available to the investigators.

6. Data collection

Clinical, laboratory and background data will be collected at the time of enrolment, during the ICU-stay, at ICU-discharge and at follow-up. Data will be obtained from hospital records,

and relatives and close friends of the patient, and will be entered into a web-based electronic case report form (eCRF) by site personnel. Data from the web-based forms will be migrated to a trial database, which will be handled by the coordinating team. The sponsor supplies a standard description of all units of measurement in the eCRF. If a trial site uses different units of measurement and this might be a potential source of error, the site investigator should contact the coordinating team to have the data capture module modified. Data not obtainable will be registered as missing and measures to obtain data should not delay intervention or concomitant treatment. All efforts will be made to collect data on the primary and secondary outcomes for patients transferred to a non-REDUSE ICU. Fluid data will be collected as long as the patient receives the intervention. A detailed description of data is provided in Appendix A

6.1 Quality control of data

Site principal investigators will be responsible for training clinical staff on correctly entering variables into the electronic case report forms (eCRFs). Particular emphasis will be placed on recording fluid administration and fluid balance data in a standardized manner. Instructions will be available in the trial master file and eCRFs. All sites will receive a site initiation visit via a digital meeting from an independent monitor before the start of inclusion. Moreover, monitors will visit all sites when 10 participants have been included. The visits will include control of routines for data collection and entry as well as quality control of data by source data verification. To minimize missing data and ensure adherence to the protocol we will use central monitoring and we will develop a central monitoring plan before the start of the trial. The site investigator must sign all eCRFs before trial completion to verify that the recorded data is correct and complete.

6.2 Biobank

We will collect blood samples at baseline, 3 days , and 5 days after randomization. At each time point we will collect 12 ml of blood. Blood samples will be processed and aliquoted according to a separate protocol. All samples will be transported to and stored in a central biobank after the trial is completed . Samples may be analysed for routine clinical laboratory measurements and prognostic biomarkers, including markers of neuronal injury, inflammation, and mitochondrial content. No analysis will occur before the trial ends, and no results from the biobank will be published in the initial manuscript. Participation in the biobank will be optional for each site.

7. Ethics and Informed consent

Ethics applications will be submitted to the National Research Ethics boards of participating countries. The applications will seek approval for a deferred consent process. This is based on the premise that to have the greatest possible impact; the intervention has to be started as soon as possible after admission to the ICU. Because cognitive symptoms are hallmark symptoms of septic shock, we regard it as impossible in most cases to obtain an informed consent at the time of presentation (Singer, 2016). We judge that this strategy is justifiable according to the Declaration of Helsinki article 30 available from the World Medical Association. Surviving participants will be asked for written consent as soon as they are able to make an informed decision. The participants will be provided with written and oral information on this trial to make an informed decision about participation in the trial. A

participant who does not give consent will be asked if already collected data can be used. The consent form must be signed by the participant. At Swedish sites, a member of the research team will approach the legal representative or a personal consultee (relative or close friend) as soon as practically possible to inform about the trial and seek their opinion about the participation of the patient in the trial.

8. Data management

8.1 Data handling and record keeping

Individual patient data will be handled as ordinary chart records and will be kept according to the legislation (e.g., data protection agencies) of each participating country. Data will be entered into a web-based electronic case report form (eCRF). The electronic data capture module fulfils criteria for handling of patient data according to the Swedish legislation on management of personal data and will be compliant with the General Data Protection Regulation of the EU (European Parliament and Council of the European Union directive 2001/20/EC) and with the Federal Drug Administration's guidelines for electronic signatures (FDA 21 CFR Part 11 Guidelines for Electronic Signatures). All original records will be retained at trial sites or at the principal investigator for 15 years to allow inspection by relevant authorities. The trial database will be maintained for 15 years.

8.2 Quality control and quality assurance

The trial will be externally monitored by national monitoring offices coordinated by the clinical trial manager and Clinical Studies Sweden, Forum South. All variables will be collected in a participant-specific trial ledger or directly in the eCRF. Site principal investigators will be responsible for training clinical staff on how to enter variables correctly. Special emphasis will be given to how to record fluid administration and fluid balance in a standardized manner. Instructions will be available in the trial ledger and in the electronic case report forms (eCRFs). All sites will have a site initiation meeting with monitors before start of inclusion and a closeout visit at end of study. Moreover, all sites will be visited by monitors when about 10 participants from that site have been included. The visits will include control of routines for data collection and data entry as well as quality control of data by comparing selected source data with data entered in eCRFs. The site investigators will be responsible for entering all relevant data into the electronic CRFs. The CRFs will be constructed in order to ensure data quality with predefined values and ranges on all data entries. To further optimize completeness and quality of data we will also use central data monitoring. A detailed central data monitoring plan will be developed before inclusion of 100 patients in the trial.

9 Adverse events

Detection, documentation and reporting of adverse events will be the responsibility of the local investigator.

9.1 Definitions

Patients with septic shock in the ICU experience a host of adverse events. Only a small number of those could be considered to be related to the intervention. In addition to the patient-centred complications described above we will monitor patients for;

- Suspected unexpected serious adverse events (SUSAE) (an adverse event not reasonably explained by other factors than the intervention which may cause death or be life threatening, prolong hospitalisation or may result in significant disability/incapacity

9.2 Reporting of SUSAEs
All SUSAEs observed by the investigator or other caregivers must be recorded in the eCRF. The circumstances of a SUSAE should be described and should be reported to the principal investigator within 48 hrs. The causality between the trial intervention and the SUSAE should be assessed by the site investigator. The local investigator is required to follow the participant until resolution of symptoms. Reports of a SUSAE will be assessed for safety by a qualified physician in the trial management team (medical monitor). SUSAEs will be reported to the DSMC within 3 days of reaching the steering group.

10. Statistical analysis plan
A detailed statistical analysis plan will be published before the randomization of the last participant. In short, data will be analysed by two independent statisticians blinded to the treatment on an intention-to-treat basis. The intention-to-treat population is defined as all randomized patients who consented to use of their data. The per-protocol population is defined as all randomized patients fulfilling all inclusion criteria and no exclusion criteria who consented to use of their data, and excluding those having one or more protocol deviations as defined above (5.5.3). Patients will be considered to be included in the trial when they are randomized.

10.1 Sample size
10.1.1 Primary outcome
The sample size calculation is based on an expected 90-day mortality of 45 % in the control group (Hjortrup 2016, Perner 2012, Hernandez 2019, Holst 2014). To detect an absolute reduction in mortality of 7.5%, with an alfa of 5% and a power of 90% the required sample size is 1808. We chose 7.5% as anticipated intervention effect because it is a realistic and clinically relevant effect size observed in previous large intensive care trials assessing interventions in critically ill patients (Perner 2012, Andersen-Ranberg 2023). To account for loss to follow-up and no-consent from some patients we will include a total of 1850 patients.

10.1.2 Secondary outcomes
Based on a minimally important relative risk reduction of 20% and a rate of complications in the control group of 30%, the sample size yields a power of 99% for the complications outcome (Meyhoff 2022).

Based on a minimally important difference of 2 days, and the data collected in the REDUSE feasibility trial (Lindén 2024), we used simulations to ascertain the power using Van Elteren as the primary analysis. Using 1,000 iterations, we found a power of 80% mechanical ventilation-free days outcome.

Based on a minimal important difference of 1.5, a standard deviation of 2.8, (Hodgson 2022) the sample size yields a power of 100% for the MOCA-BLIND score outcome.

Based on a minimal important difference of 5 points, a standard deviation of 20 points, the sample size yields a power of 99% for the EQ-VAS score outcome (Contrin 2013, Granja 2004, Hammond 2020).

10.2 Analysis methods

Analyses will be performed according to an intention-to-treat principle. All analyses will be adjusted for site of admission. All analyses will be adjusted for site of admission. The primary outcome and the first secondary outcome (complications) will be analysed using mixed effects logistic regression with site as a random intercept. RR will be estimated using the 'nlcom' STATA command or G-computation in R (R Core Team, Vienna, Austria). Count data (mechanical ventilation-free days) will be analysed by van Elteren test with site as a covariate. Secondary continuous outcomes (MoCA-BLIND and EQ-VAS) will be analysed using mixed effects linear regression with site as a random intercept.

All primary conclusions will be based on our primary outcome and, a priori, secondary and explorative outcome results will be considered as hypothesis generating. Based on this we will consider a P-value of 0.05 as a threshold for statistical significance for all analyses.

All primary conclusions will be based on our primary outcome and, a priori, secondary and exploratory outcome results will be considered as hypothesis generating. Based on this we will consider a P-value of 0.05 as a threshold for statistical significance for all analyses.

10.3 Missing data

Missing data will be handled according to the recommendation by Jakobsen et al. 2017. We anticipate that the proportion of missing values on primary and secondary outcomes other than health-related quality of life and cognitive function will be less than 5%. For health-related quality of life and cognitive function we expect missing data to be less than 15%.

10.4 Subgroup analysis

The heterogeneity of the intervention effect on the primary and secondary outcomes will be assessed in subgroups based on baseline characteristics at randomization.

- Mechanical ventilatory support at the time of randomization (defined as invasive or non-invasive mechanical ventilation or nasal high flow treatment [yes/ no]).
- Acute kidney injury at the time of randomization (≥ KDIGO stage 1 [yes/ no])
- Sex (male/ female)
- Age (≥ 65 years [yes/ no])
- Frailty Clinical frailty scale (≤ 3, 4, >4).
- Weight at admission (≥ median of the intention to treat population [yes/ no])
- Validated infection as suggested by a Linder-Mellhammar score ≥ 3 (yes/ no) (Mellhammar 2022)
- IV fluid at time of randomization < 30 ml/kg (yes/ no)

- Plasma Lactate (> 4 mmol/l) (yes / no)

10.4 Exploratory analyses

We will perform an analysis including only centres in which a median difference in total volume of administered fluid between the groups in is > 3L at day 4. We will also perform a subgroup analysis including only centres in which the median difference in cummulative fluid balance is > 2 L at day 4. Because patients staying longer in the ICU will be exposed to the intervention for a longer time will perform a subgroup analysis of patients staying four days or more in the ICU. Because the amount of glucose may differ between the intervention- and control group we will also perform an analysis of the interaction between the amount of intravenous glucose prior to full enteral nutrition or initiation of parenteral nutrition and treatment effect.

10.5 Statisticians

Analyses of results will be performed by two independent statisticians.

10.6 Data safety monitoring committee

There will be an independent Data Safety Monitoring Committee (DSMC) which will monitor the trial according to the charter for the DSMC (Appendix B). The DSMC will arrange with an independent statistician to conduct a blinded interim analysis. The DSMC will be able to request unblinding of data if they find it necessary. The DSMC will be provided with data on survival and can initiate an analysis at any time they request. Lan-DeMets group sequential monitoring boundaries will be used to decide if the trial should be stopped (Lan and Demets, 1983).

10.7 Members of the data safety monitoring committee

Todd W. Rice, MD, MSc Associate Professor of Medicine, Division of Allergy, Pulmonary, and Critical Care Medicine, Nashville, TN, USA

Naomi Hammond, PhD RN BN MN (Crit. Care) MPH Senior Research Fellow/ Operations Lead, Critical Care Program Intensive Care Clinical Research Manager, The George Institute for Global Health, Sidney, Australia

Qiang Li, MBiostat BPH AStat, Lead Biostatistician, Biostatistics and Data Science Division Stats lead, Global Brain Health Initiative, Conjoint Senior Lecturer, Faculty of Medicine, UNSW and the The George Institute for Global Health Sydney, Australia.

10.8 Environmental analysis

In addition to climate impacts, other environmental impacts of the intervention will be estimated using life cycle assessment methodology (Hauschild 2018). A separate detailed protocol outlining the boundaries of the climate- and environmental analysis, the impact assessment methodology and statistical analysis plan will be submitted for publication before the last patient is randomized.

10.9 Health economic analysis

We will analyse health economic aspects of the intervention. A detailed plan for such an analysis will be published before the last participant is randomized.

11. Publication of Data

The analysis process will be performed with the allocation code unbroken and with the trial arms only known as A and B. Two abstracts of the main publication will be prepared before the allocation code is broken, with the different arms interchanged (one assuming arm A is the intervention arm, and the other assuming arm B is the intervention arm). All authors must approve both versions before the code is broken. The final main publication will be submitted to a peer-reviewed international journal. Authorship will be granted using the Vancouver definitions and depending on personal involvement and fulfilment of the authors' respective roles. The author list will include the steering group members, national investigators and additional names. Centres recruiting >30 participants will be entitled to one name, >60 two names, >100 three names, >150 four names, >220 five names in the author list (additional names). After the author list, there will be added: "and the REDUSE-trial group" and a reference to an appendix with all sites, site investigators and the number of participants enrolled. The main publication will report the primary and secondary outcomes. Exploratory outcomes will, due to complexity of reporting be submitted to a peer-reviewed journal in separate manuscripts, as will the studies based on the biobank. Ideas for additional substudies will be presented to the steering group. The steering group will decide which substudies to prioritize. No substudy will be published prior to publication of the main article.

11.1 Data sharing

Approximately one year after publication of the main report of this trial individual de-identified data will be available for sharing with researchers who provide a methodologically sound proposal as judged by the steering committee. To gain access, data requestors will need to sign a data access agreement.

12. Insurance

When pre-existing insurance is not available, indemnity to meet the potential legal liability of investigators/collaborating hospitals for harm to participants arising from the conduct of the research will be provided by the REDUSE-trial through the sponsor: Region Skåne. The insurance for each country will be specified in each site agreement before the commencement of patient inclusion at that site.

13. Funding

The trial will be funded by non-commercial foundations for medical research. Patient recruitment will not commence until there is sufficient funding to allow for inclusion and follow-up of the proposed sample size.

14. Timeline

14.1 One year following the start of the project (end of 2024).
Run in period by the end of which more than 10 sites should have received training in follow-up evaluations, received a site initiation visit from monitors and started to include patients.

14.2 Two years following the start of the project (end of 2025).
- A total of 600 patients should have been included.
- Sites with more than 10 included patients should have received a monitoring visit.

14.3 Three years following the start of the project (end of 2026).
- A total of 1200 patients should have been included by the end of the year
- The interim analysis of the first 400 patients with 90-day outcome data should have been completed.

14.4 Four years following the start of the project (end of 2027).
A total of 1850 patients should have been included by the end of the year

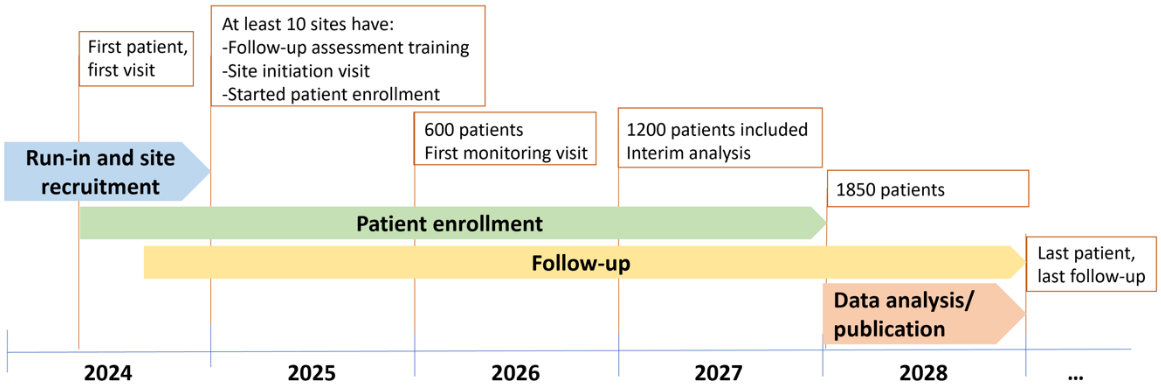

15. Investigators

15.1 Management group
Peter Bentzer, MD, PhD principal investigator
Anja Lindén, MD, PhD coordinating investigator
Niklas Nielsen, MD, PhD senior investigator
Janus Christian Jakobsen, MD, PhD chief trialist
Gisela Lilja, OT, PhD, follow-up coordinator
Jane Fisher, PhD, trial coordinator
Markus Harboe Olsen, MD, PhD, statistical advisor
Matt Wise, MD, PhD, national investigator in the UK.
Markus Skrifvars, MD, PhD, national investigator in Finland
Pirkka T. Pekkarinen, MD, PhD, co-national investigator Finland

15.2 Steering group
All principal site investigators will be part of the steering group. A representative from the newly formed patient organisation "Sepsisföreningen" will be invited to the management group meetings if/when aspects of the conduct of the trial which are deemed to be of importance from a patient perspective are discussed. Such aspects include any change in the protocol with ethical implications.

References

Andersen-Ranberg NC, Poulsen LM, Perner A, et al Haloperidol for the Treatment of Delirium in ICU Patients. *N Engl J Med.* 2022; 387:2425–2435.

Boyd JH, Forbes J, Nakada TA, et al. Fluid resuscitation in septic shock: a positive fluid balance and elevated central venous pressure are associated with increased mortality. *Crit Care Med.* 2011; 39:259-265.

Chen C, Kollef MH. Targeted Fluid Minimization Following Initial Resuscitation in Septic Shock: A Pilot Study. *Chest*. 2015; 148: 1462-1469.

Contrin LM, Paschoal VD, Beccaria LM, et al. Quality of life of severe sepsis survivors after hospital discharge. *Revista Latino-americana de Enfermagem*. 2013; 21: 795-802.

Douglas IS, Alapat PM, Corl KA, et al. Fluid Response Evaluation in Sepsis Hypotension and Shock: A Randomized Clinical Trial. *Chest*. 2020; 158: 1431-1445.

Evans L, Rhodes A, Alhazzani W, et al. International Guidelines for Management of Sepsis and Septic Shock 2021. *Crit Care Med*. 2021; 49: e1063-e1143.

Finfer S, Liu B, Taylor C, et al. Resuscitation fluid use in critically ill adults: an international cross-sectional study in 391 intensive care units. *Crit Care*. 2010; 14: R185.

Granja C, Dias C, Costa-Pereira A, Sarmento A. Quality of life of survivors from severe sepsis and septic shock may be similar to that of others who survive critical illness. *Crit Care* 2004; 8: R91-8.

Hammond NE, Finfer SR, Li Q, et al. ADRENAL Trial Investigators and the Australian and New Zealand Intensive Care Society Clinical Trials Group. Health-related quality of life in survivors of septic shock: 6-month follow-up from the ADRENAL trial. *Intensive Care Med*. 2020; 46: 1696-1706.

Hauschild MZ, Rosenbaum RK, Olsen SI, editors. Life cycle assessment: theory and practice. Cham: Springer; 2018. https://doi.org/10.1007/978-3-319-56475-3

Hernández G, Ospina-Tascón GA, Damiani LP, et al. Effect of a Resuscitation Strategy Targeting Peripheral Perfusion Status vs Serum Lactate Levels on 28-Day Mortality Among Patients with Septic Shock: The ANDROMEDA-SHOCK Randomized Clinical Trial. *JAMA.* 2019; 321: 654-664.

Hjortrup PB, Haase N, Bundgaard H, et al. Restricting volumes of resuscitation fluid in adults with septic shock after initial management: the CLASSIC randomised, parallel-group, multicentre feasibility trial. *Intensive Care Med.* 2016; 42:1695-1705.

Hodgson CL, Higgins AM, Bailey M, et al. Comparison of 6-month outcomes of sepsis versus non-sepsis critically ill patients receiving mechanical ventilation. *Crit Care* 2022; 26: 174.

Holst LB, Haase N, Wetterslev J, et al. Lower versus higher hemoglobin threshold for transfusion in septic shock. *N Engl J Med*. 2014; 371: 1381-1391.

Iwashyna TJ, Ely EW, Smith DM, et al. Long-term cognitive impairment and functional disability among survivors of severe sepsis. *JAMA*. 2010; 304: 1787-94.

Jakobsen JC, Gluud C, Wetterslev J, et al. When and how should multiple imputation be used for handling missing data in randomised clinical trials - a practical guide with flowcharts. *BMC Med Res Methodol* 2017, 17: 162.

Kellum JA, Lameire N, KDIGO AKI Guideline Work Group. Diagnosis, evaluation, and management of acute kidney injury: a KDIGO summary (Part 1). *Crit Care.* 2013; 17: 204.

Kjær MN, Meyhoff TS, Sivapalan P, et al. Long-term effects of restriction of intravenous fluid in adult ICU patients with septic shock. *Intensive Care Med*. 2023; 49: 820-830.

Lan G, DeMets D. Discrete Sequential Boundaries for Clinical Trials
Biometrika, 1983, 70; 659-663

Lindén-Søndersø A, Jungner M, Spångfors M, et al. Survey of non-resuscitation fluids administered during septic shock: a multicenter prospective observational study. *Ann. Intensive Care*. 2019; 9: 132.

Lindén A, Fisher J, Lilja G, et al. Protocolised reduction of non-resuscitation fluids versus usual care in patients with septic shock (REDUSE): a protocol for a multicentre feasibility trial. *BMJ Open.* 2023; 13:e065392.

Lindén A, Spångfors M, Olsen MH, et al. Reduction of non-resuscitation fluids in septic shock: a Randomized Clinical Feasibility Trial. *Crit Care*. 2024;28:166.

Marik PE, Linde-Zwirble WT, Bittner EA, et al. Fluid administration in severe sepsis and septic shock, patterns and outcomes: an analysis of a large national database. *Intensive Care Med*. 2017; 43: 625-632.

McMillan T, Wilson L, Ponsford J, et al. The Glasgow Outcome Scale - 40 years of application and refinement. *Nat Rev Neurol.* 2016; 12: 477-85.

Mellhammar L, Elén S, Ehrhard S, et al. New, Useful Criteria for Assessing the Evidence of Infection in Sepsis Research. *Crit Care Explor*. 2022; 4: e0697.

Meyhoff TS, Hjortrup PB, Wetterslev J, et al. Restriction of Intravenous Fluid in ICU Patients with Septic Shock. *N Engl J Med*. 2022; 386: 2459-2470.

Mikkelsen ME, Christie JD, Lanken PN, et al. The Adult Respiratory Distress Syndrome Cognitive Outcomes Study. *Am J Respir Crit Care Med*. 2012; 185:1307–1315.

Needham DM, Sepulveda KA, Dinglas VD, et al. Core Outcome Measures for Clinical Research in Acute Respiratory Failure Survivors. An International Modified Delphi Consensus Study. *Am J Respir Crit Care Med*. 2017; 196: 1122-1130.

Pendlebury ST, Welch SJ, Cuthbertson FC, et al. Telephone assessment of cognition after transient ischemic attack and stroke: modified telephone interview of cognitive status and telephone Montreal Cognitive Assessment versus face-to-face Montreal Cognitive Assessment and neuropsychological battery. *Stroke* 2013; 44: 227–229.

Perner A, Haase N, Guttormsen AB, et al. Hydroxyethyl starch 130/0.42 versus ringer's acetate in severe sepsis. *N Engl J Med.* 2012; 367: 124-134.

Rudd KE, Johnson SC, Agesa KM, et al. Global, regional, and national sepsis incidence and mortality, 1990-2017: analysis for the Global Burden of Disease Study. *Lancet*. 2020; 395: 200-211.

Sakr Y, Rubatto Birri PN, Kotfis K, et al. Higher fluid balance increases the risk of death from sepsis: results from a large international audit. *Crit Care Med.* 2017; 45: 386–94.

Silversides JA, Fitzgerald E, Manickavasagam US, et al. Deresuscitation of patients with iatrogenic fluid overload is associated with reduced mortality in critical illness. *Crit Care Med.* 2018; 46: 1600-1607.

Singer M, Deutschman CS, Seymour CW, et al. The Third International Consensus Definitions for Sepsis and Septic Shock (Sepsis-3). *JAMA*. 2016; 315: 801–810.

Sivapalan P, Ellekjaer KL, Jessen MK, et al. Lower vs Higher Fluid Volumes in Adult Patients With Sepsis: An Updated Systematic Review With Meta-Analysis and Trial Sequential Analysis. *Chest.* 2023; 164: 892-912.

Téllez N, Río J, Tintoré M, Nos C, et al. Does the Modified Fatigue Impact Scale offer a more comprehensive assessment of fatigue in MS? *Mult Scler*. 2005; 11: 198-202.

van Reenen M, Janssen B. EQ-5D-5L user guide: basic information on how to use the EQ-5D-5L instrument. Rotterdam: *EuroQol Research Foundation* 2015; 9.

Wittich W, Phillips N, Nasreddine ZS, et al. Sensitivity and specificity of the Montreal Cognitive Assessment modified for individuals who are visually impaired. *J Visual Impairm Blindness* 2010; 104: 360–368.

Üstün TB, Kostanjsek N, Chatterji S, et al. Eds. Measuring Health and Disability: Manual for WHO Disability Assessment Schedule (WHODAS 2.0). *World Health Organization* 2010

Table 1. Study Schedule

	Study day[a]								
	**1**	**2**	**3**	**4**	**5**	**6- ICU dis.[b]**	**90**	**180**	**365**
	**ENROLMENT**								
**Eligibility assessment**	x								
**Informed consent**	x	x	x	x	x	x			
**Randomization**	x								
	**INTERVENTION**								
**Protocolized administration of non-resuscitation fluids**	x	x	x	x	x	x			
	**ASSESSMENTS**								
**Mortality**							x	x	x
**Complications**	x	x	x	x	x	x			
**Mechanical ventilation-free days**							x		
**Cognitive function**								x	
**Health related quality of life**								x	
**Hospital-free days**							x		
**Vasopressor-free days**							x	x	
**RRT-free days**							x		
**MAKE**							x		
**Dose of diuretics**					x				
**Glasgow Outcome Scale Extended**								x	
**WHO Disability Assessment Schedule**								x	
**Modified Fatigue Impact Scale**								x	
**Number of days in the ICU**							x		
**Hypoglycaemia, Hypernatraemia, Acid-base disturbances**	x	x	x	x	x	x			
**CVC related complications**	x		x		x				

a) Day 1 one is defined as the day of inclusion.
b) Intervention will only be administered while the patient is in an REDUSE-ICU. If patient is transferred to a non-REDUSE ICU she/he will be considered as discharged from ICU.
RRT= Renal Replacement therapy: MAKE = Major adverse kidney event: CVC = central venous catheter

Appendix A. Description of collected data

During screening

- Initials (Initials starting with the first name. E.g. James Tiberius Kirk = JTK)
- Age (years, calculated from date of birth)
- Sex at birth (F/M)
- Lactate (mmol/L; highest value at any time while the patient is in the ICU and receiving vasopressors)
- Variables related to inclusion criteria
    - Adult (age ≥18 years) (yes/no)
    - Septic shock (yes/no) (while in the ICU but no later than 12 hours after ICU admission, defined as: Confirmed or suspected infection AND Need for vasopressor therapy to maintain mean arterial pressure ≥ 65 mmHg despite adequate fluid resuscitation (≥ 1 L resuscitation fluid within the last 12 hours) regardless of whether the need for vasopressor arose in connection with sedation AND Lactate > 2 mmol)
    - Currently receiving vasopressors (yes/no)
    - Lactate (Highest value at any time while the patient is in the ICU and receiving vasopressors) (A patient may be included even if lactate is ≤ 2 at the time of inclusion as long as all of the following are true: The patient needs vasopressors at inclusion AND The patient has had a lactate >2 and simultaneous need for vasopressor at any time while in the ICU) (mmol/L or mg/dL)
- Variables related to exclusion criteria
    - Date and time of ICU admission (dd-mmm-yyyy, hh:mm)
    - Confirmed or suspected pregnancy (yes/no)
    - Patient previously included in the REDUSE trial (yes/no)

Consent information

- Patient informed (Y/N) and date (dd-mmm-yyyy)
- Reason not informed (if N)
- Patient consented (Y/N)
    -Patient granted use of data already collected (Y/N)
- Consent withdrawn (Y/N)
- Date of withdrawal (dd-mmm-yyyy)
- Can the data be used (Y/N)
- Can primary outcome (mortality) be collected (Y/N)

Background data

- Height (cm; Sites using imperial measurements: Record estimated height in feet and inches)
- Weight at baseline (kg, standardized according to local practice; Record the weight measurement taken closest to time of inclusion; Sites using imperial measurements: Record estimated weight in pounds (lbs)
- Body mass index BMI (kg/cm^2)
- Clinical Frailty Score (Patient's estimated level of fitness/frailty prior to hospital admission; Defined according to: https://www.dal.ca/sites/gmr/our-tools/clinical-frailty-scale.html)
- Baseline creatinine [lowest in the 12 months preceding randomization] (µmol/L)

- Charlson Comorbidity Index (Charlson et al. J Chronic Dis. 1987; 40: 373-83)
- Type of initial antimicrobial treatment
- Suspected pathogen(If culture was taken, indicate the culture result)
    - Suspected pathogen sensitive to initial antibiotic treatment (sensitive/intermediate /resistant/not available)
- Hospital admission (dd-mmm-yyyy, hh:mm) (Date and time that the patient was admitted to hospital they were randomised at. If the patient was transferred from another hospital, record the date and time of admission to the hospital where the patient was randomised.)
- Hospital location prior to randomization a) Emergency department, b) Operating room, c) Other ICU, d) Other unit
- Steroid treatment prior to hospital admission (Y/N)
- Surgery prior to inclusion/randomization (Y/N), if yes, specify: a) Head and neck, b) Thorax, c) Abdominal/pelvic, d) Extremities, e) Trauma f) Other
- Origin of sepsis (Mellhammar et al. Crit Care Exp 2022; 4: e0697)

Baseline variables at study inclusion (values closest in time to enrolment, within ± 6 h, unless other timeframe is specified; lab values either arterial or venous.)

- Body temperature (degrees Celsius)
- SAPS-III (Simplified acute physiology score-III) (Moreno et al. Intensive Care Med. 2005;31:1345-55)
- Glasgow Coma Score (GCS) (Teasdale and Jennett B. Lancet. 1974; 2: 81-4)
- Creatinine (µmol/L)
- Renal replacement therapy (Y/N)
- Bilirubin (µmol/L)
- Platelet count (x10^9/ml)
- Mean arterial pressure (mmHg)
- Systolic pressure (mmHg)
- Type of inotropic drug or vasopressor (any dose of dobutamine, dopamine, vasopressin or other V1A agonists, levosimendan, angiotensin II, noradrenaline, adrenaline, milrinone, or other)
- Noradrenaline dose (highest dose in the 6 hours prior to enrollment; µg/kg/min)
- Corticosteroid treatment (Y/N)
- Atrial fibrillation/flutter (Y/N)
- Ischemic events (Y/N) (criteria described above), if yes, specify: a) Limb, b) Cerebral, c) Heart, d) Intestine
- Heart rate (bpm)
- Ventilatory support (nasal catheter, nasal high flow oxygen, Hudson mask or similar, reservoir mask, non-invasive mechanical ventilation, invasive mechanical ventilation [defined as mechanical ventilation through an orotracheal tube or through a tracheostomy], none. Classification at each day will be based on the highest level of support,
- CRP (g/L)
- Leucocytes (x10^9 cells/L)
- Haemoglobin (g/L)
- Potassium (mmol/L)

- Sodium (mmol/L)
- Chloride (mmol/L)
- Blood glucose (mmol/L)
- Arterial blood gas on inclusion (Y/N)
- FiO2 (%)
- PaO2 (kPa)
- PaCO2 (kPa)
- pH
- Base excess (BE, mEq/L)
- Volume of fluid intake in the 24h prior to inclusion (Crystalloids (Ringer's acetate/lactate [ml], 0.9% NaCl [ml], other [ml]); Colloids (Albumin 4-5% [ml], Albumin 20% [ml], other [ml]); Blood products (Erythrocytes [ml], Plasma [ml]- Platelets [ml]); Glucose (any concentration [ml]); Parenteral nutrition (ml); Enteral nutrition (ml); Enteral water (ml))

Daily from day 1 - 5 after inclusion

- Patient in a REDUSE ICU this day (Y/N)
- Resuscitation fluids (Crystalloids administered to correct hemodynamic impairment as noted in the patient chart or given at a rate > 5 ml/kg/h (Ringer's acetate/lactate [ml], 0.9% NaCl [ml], other [ml]; Colloids (Albumin 4-5% [ml], Albumin 20% [ml], other [ml]; Blood products (Erythrocytes [ml], Plasma [ml], Platelets [ml]).
- Intravenous vehicles and drugs (Antibiotics [mL], Inotropes (includes dobutamine, levosimedan, or dopamine <5mcg/kg/min) [mL], Vasopressors [mL], Analgesics [mL], Sedatives [mL], Insulin [mL] and dose [E/24h; If the patient was in the ICU for less than 24 hours this day, extrapolate the dose to 24 hours], Potassium [mL], Other electrolytes [mL], Other drugs [mL], 5% glucose used as a vehicle [mL], Other concentration of glucose used as a vehicle [mL and concentration in %]).
- Maintenance/replacement and nutrition (Crystalloids administered for reasons other than correcting hemodynamic impairment. If indication in the medical charts is unclear, crystalloids at a rate below 5 ml/kg/h will be classified as maintenance fluids (Ringer's acetate/lactate [ml], 0.9% NaCl [ml], other (ml)), Glucose (2.5% [ml], 5% [ml], 10% [ml], 20% (ml), other glucose strength (mL and concentration in %)), was no or 20% glucose given in accordance with established local protocols/routines; Parenteral nutrition (ml); Enteral nutrition (ml); Enteral water (ml); Other fluids (mL); Total caloric intake [including Propofol and glucose solutions] (kcal)
- Diuretics (Loop diuretics/furosemide [mg/24h], Other (type of drug and mg/24h)
- Fluid output (Urinary output [ml], Drains [ml], Haemorrhage [ml], Faeces [if liquid and collected through a faecal management system, ml], Fluid removal in RRT [ml], Other losses [evaporation excluded] (ml)
- Fluid balance (Total fluids that day [mL], Fluid balance [mL], Cumulative fluid balance [mL], Total volume of resuscitation fluids [mL], Total volume of non-resuscitation fluids [mL], Weight [kg], Fluid balance goal for next 24h [Y/N, and volume in mL])
- Creatinine [highest](µmol/L)
- Acute Kidney Injury (Calculated based on creatinine and urine output [Y/N], if yes specify KDIGO score)
- Renal replacement therapy (Y/N)
- Earliest urea (mmol/L; Value closest in time to the change of day at the institution.)
- Lowest MAP (mmHg; Lowest value that day)

- Type of inotropic drug or vasopressor (any dose of dobutamine, dopamine, vasopressin or other V1A agonists, levosimendan, angiotensin II, noradrenaline, adrenaline, milrinone, or other)
- Noradrenaline dose (highest dose during the day; µg/kg/min)
- Corticosteroid treatment (Y/N)
- Atrial fibrillation/flutter (Y/N)
- Mechanical ventilation (Y/N)
- Lowest PaO2 (kPa)
- FiO2 (at time of lowest PaO2; %)
- Lactate [highest] (mmol/L)
- Sodium [earliest] (mmol/L)
- Potassium [earliest] (mmol/L)
- Chloride [earliest] (mmol/L)
- Blood glucose [earliest] (mmol/L)
- Ischemic events (Y/N) (criteria described above), if yes, specify: a) Limb, b) Cerebral, c) Heart, d) Intestine
- Hypoglycemia (Mild/moderate hypoglycaemia [2.3 - 3.9 mmol/L] (Y/N), Severe hypoglycaemia [≤ 2.2 mmol/L] (Y/N)
    - If yes to severe hypoglycemia:
        - Was the patient receiving IV glucose at the time of the event (Y/N)
        - Was the patient receiving insulin at the time of the event? (Y/N)
        - Was the patient receiving enteral nutrition at the time of the event (Y/N)
        - Additional information/details (free text)
- Hypernatremia [>159 mmol/L] (Y/N)
- Hyperchloremic acidosis [pH<7.15 and plasma-chloride >115 mmol/L] (Y/N)
- Metabolic alkalosis [pH>7.59 and base excess >9] (Y/N)
- Central venous catheter complications (Includes malfunctions, infections, thrombosis and venous stenosis) (Y/N and specify)
- Suspected unexpected adverse events (SUSAE) (Y/N); (A SUSAE is an adverse event not reasonably explained by other factors than the intervention which may cause death, be life threatening, prolong hospitalisation, or result in significant disability/incapacity.)

Daily from day 6 to discharge from REDUSE ICU or day 90

- Patient in a REDUSE ICU this day (Y/N)
- Fluids (Total volume of resuscitation fluids [mL], Total volume of non-resuscitation fluids [mL], Total fluid output [mL], Fluid balance[mL])
- Ischemic events (Y/N) (criteria described above), if yes, specify: if yes, specify: a) Limb, b) Cerebral, c) Heart, d) Intestine
- Any new Acute kidney injury (Y/N, if yes specify KDIGO score [1-3])
- Hypoglycemia (Mild/moderate hypoglycaemia [2.3 - 3.9 mmol/L] (Y/N), Severe hypoglycaemia [≤ 2.2 mmol/L] (Y/N)
- Hypernatremia [>159 mmol/L] (Y/N)
- Hyperchloremic acidosis [pH<7.15 and plasma-chloride >115 mmol/L] (Y/N)
- Metabolic alkalosis [pH>7.59 and base excess >9] (Y/N)
- Central venous catheter complications (Includes malfunctions, infections, thrombosis and venous stenosis) (Y/N and specify)
- Suspected unexpected adverse events (SUSAE) (Y/N))

At discharge from REDUSE ICU

- ICU discharge (If patient is transferred to a non-REDUSE ICU they will be considered as discharged from the ICU. If the patient is transferred to a REDUSE ICU, this will not be considered an ICU discharge.)
  - Date and time of ICU discharge (dd-mmm-yyyy, hh:mm)
  - Status at ICU discharge (alive/deceased)
  - Length of last ICU stay (hours/days)
  - Length of ICU stay (hours/days)

-ICU readmission
  - Date and time of ICU readmission and discharge (dd-mmm-yyyy, hh:mm)
  - Status at ICU discharge (alive/deceased)

- Withdrawal of life sustaining therapies (WLST) (Including any withdrawal of care which results in the patient not receiving the intervention, for example, palliation.) (Y/N), if yes, specify reason:
  - Irreversible organ failure (Y/N); if yes specify Cardiac, Lung, Liver, Kidney, Coagulation, Brain or Other
  - Medical comorbidity (Y/N)
  - Other (Y/N); specify
  - Date and time when WLST decision was made (dd-mmm-yyyy, hh:mm)

Up to 90 days after inclusion

- Date of follow-up (dd-mmm-yyyy)
- Status (alive/deceased)
- Days alive and free of renal replacement therapy (RRT) (days; Only include full days without RRT. The data should be recorded for up to 90-days regardless of how the patient was discharged or readmitted.)
- Non-invasive and invasive mechanical ventilation-free days (days; Only include full days without invasive or non-invasive mechanical ventilation. Nasal high flow oxygen therapy is not considered mechanical ventilation. The data should be recorded for up to 90-days regardless of how the patient was discharged or readmitted)
- Vasopressor-free days (days) (Only include full days without vasopressors. The data should be recorded for up to 90-days regardless of how the patient was discharged or readmitted.)
- Days spent in any ICU (days) A day with any time spent in any (incl. non-REDUSE) ICU counts as an ICU day, even if it is less than 24 hours. The data should be recorded for up to 90-days regardless of how the patient was discharged or readmitted.)
- Hospital-free days (days; Only count full days out of hospital. Time spent in a rehabilitation facility, long-stay hospital, hospice, or nursing home is considered as time spent out of hospital. Includes any hospital (even non-REDUSE sites). The data should be recorded for up to 90-days regardless of how the patient was discharged or readmitted)
- ICU-free days (days)
- Creatinine at hospital discharge (µmol/L; Latest value recorded within 90 days at any hospital)

At 6 months

- Date of follow-up (dd-mmm-yyyy)
- Status (alive/deceased)
- Was follow-up completed; if no specify reason (Patient lost to follow-up, Alive but refused to consent to follow-up, Alive but unable to follow-up for other reasons)
- Place of follow up (Institution/ home of patient/ telephone/ digital)
- Montreal Cognitive Assessment (MoCA-BLIND) (telephone). The MoCa blind assess attention and concentration, memory, language, conceptual thinking, calculations and orientation.
- Health-Related Quality of Life (HRQoL) using the European Quality of Life visual analogue scale (EQ-VAS). EQ-VAS is a self-report of overall health and part of the EQ -5 Dimensions 5 Levels (EQ-5D5L) questionnaire and ranges from 0 to 100.
- HRQoL using the EQ-5D-5L questionnaire. EQ-5D-5L assesses HRQoL in five dimensions (mobility, self-care, usual activities, pain, discomfort and anxiety/depression) where each dimension is rated on a five-levels scale.
- World Health Organization Disability Assessment Schedule 2.0 (WHODAS). WHODAS 2.0 is a patient- or proxy-reported measure of disabilities in 12 aspects of daily life. Each question is answered on a five-level scale.
- Life Satisfaction (ranges from 1-10)
- Background information
    - Does the patient have a native language other than the test language (Y/N); if yes: judgement about whether the patient is sufficiently good at the test language to complete the tests or need interpreter
    - Problem with capabilities that may interfere with the patient's ability to perform the tests (Not including problems with hearing or vision that are corrected for by hearing aid or glasses)
        - No problems
        - Hearing
        - Vision
        - Speech problems
        - Dyslexia
        - Paresis
        - Memory problems or other cognitive problems prior to the episode of sepsis
        - Other
    - Known neurological disease (Y/N; specify) (includes Stroke, Multiple Sclerosis, Parkinson’s disease, Dementia etc.)
    - Highest education level (functional equivalent of the indicated options for a given society based on international standard classification of education by UNESCO)
        - No formal education
        - Incomplete primary/lower secondary school
        - Complete primary/lower secondary school
        - Incomplete upper secondary school
        - Complete upper secondary school
        - Some university-level education, without degree
        - University-level education, with degree

  - Marital status (married/living together as married, living together with parents/children, or living alone)
  - Current place of residence
    - Home
    - Hospital
    - Rehabilitation centre
    - Nursing home
    - Other
  - Occupational status before the episode of sepsis
    - Working full-time
    - Working part-time
    - Unemployed
    - Retired due to age
    - Retired due to disability / health problems
    - On sick leave
    - Other (e.g., student, housewife)
  - Occupational status at the time of the follow-up
    - Working full-time
    - Working part-time
    - Unemployed
    - Retired due to age
    - Retired due to disability / health problems
    - On sick leave
    - Other (e.g., student, housewife)
    - Date of return-to-work (if applicable)
  - Rehabilitation after the episode of sepsis
    - None
    - Inpatient rehabilitation
    - Outpatient rehabilitation
    - Home-based rehabilitation (community)
    - Physiotherapist only
    - Occupational therapist only
    - Counselling (by e.g., social worker or psychologist)
    - Cognitive Behavioural Therapy
    - Other
- Glasgow Outcome Scale Extended (GOSE) modified for use after Sepsis. GOSE is a clinician-reported global functional outcome scale with 8 ordinal categories, ranging from dead (1) to upper good recovery (8) based on a structured interview with the patient, proxy (e.g., a relative or close friend) or both
- Modified Fatigue Impact Scale (MFIS). MFIS is a patient-reported measure of fatigue and consists of 21 questions. Each question is answered on a five-level scale.
- Patient Questionnaire regarding scope, completeness, and timing of follow up. The questions are answered on a five-grade Likert scale or as yes/no
  - How do you feel the questions have allowed you to describe your experiences after sepsis
  - Do you feel that any questions are missing about symptoms/difficulties which affect your everyday life post sepsis

- Which is your overall experience of the follow-up
- How would you describe the timing of the follow-up
- Have you been offered a similar follow-up after your episode of sepsis from healthcare

At 1 year

- Date of follow up (dd-mmm-yyyy)
- Survival status at 1 year (alive/deceased/unknown)

Death
Date and time of death (dd-mmm-yyyy, hh:mm)

Appendix B. Charter for the Data Safety and Monitoring Committee (DSMC)

1. Introduction

This Charter defines the primary responsibilities of the DSMC, its relationship with other trial components, its membership, and the purpose and timing of its meetings. The Charter will also provide the procedures for ensuring confidentiality and proper communication, and the statistical monitoring guidelines to be implemented by the DSMC.

2. Primary responsibilities of the DSMC

The DSMC will, jointly with the steering group, be responsible for safeguarding the interests of trial participants and assessing the safety and efficacy of the interventions during the trial. The DSMC will recommend stopping or continuing the trial to the steering group of the REDUSE-trial. The steering group will be responsible for promptly reviewing the DSMC recommendations, deciding whether to continue or terminate the trial and determining whether amendments to the protocol or changes in trial conduct are required. The DSMC is planned by protocol to meet (physically or online) to evaluate the planned interim analysis of the REDUSE-trial. A statistician selected by the DSMC will perform the interim analyses. The recommendations of the DSMC regarding stopping, continuing, or changing the design of the trial should be communicated without delay to the steering group. The steering group has the responsibility to inform all investigators participating in the trial of the recommendation of the DSMC and the subsequent steering group decision as soon as possible and no later than 48 hours after the decision is made.

3. Members of the DSMC

The DSMC is an independent multidisciplinary group consisting of clinicians and a biostatistician that, collectively, have experience in managing ICU patients and in conducting, monitoring and analysing clinical trials.

3.1 Members of the DSMC

Todd W. Rice, MD, MSc Associate Professor of Medicine, Division of Allergy, Pulmonary, and Critical Care Medicine, Nashville, TN, USA

Naomi Hammond, PhD RN BN MN (Crit. Care) MPH Senior Research Fellow/ Operations Lead, Critical Care Program Intensive Care Clinical Research Manager, The George Institute for Global Health, Australia

Qiang Li, Senior biostatistician, The George Institute for Global Health, Sidney, Australia

4 Conflicts of interest

DSMC membership has been restricted to individuals free of financial, scientific, or regulatory conflicts of interest. Any DSMC members who develop significant conflicts of interest during the trial should resign from the DSMC. DSMC membership is to be for the duration of the clinical trial. If any members leave the DSMC during the trial, the steering group will appoint their replacement(s).

5. Meetings of the DSMC

The members of the DSMC will have one 'Formal Interim Analysis' meeting to review data relating to treatment efficacy, patient safety, and quality of trial conduct when 90-day follow-up data of 400 patients have been obtained. For details concerning this analysis please see sections 9 and 10 in this charter.

Based on the results of this first interim analysis, it is the DSMC's responsibility to decide if and when additional interim analyses are warranted. The DSMC may additionally meet whenever they decide and contact each other by telephone or e-mail to discuss evolving information from the trial.

6. Communication between the steering group and the DSMC

The DSMC will be blinded in its safety and efficacy data assessments. Data transferred to the DSMC will be aggregated according to treatment group and labelled as group A and group B. The DSMC can request unblinding from the steering group. The data transferred from the steering group to the DSMC will be prepared by an independent liaison, with assistance from the trial statistician, in a manner that allows all trial personnel to remain blinded.

The DSMC can request additional reports concerning outcome measures and complications at any time during the trial. The reports should be up-to date should be provided to DSMC within 1 week of the request.

7. Minutes of the DSMC meetings

The DSMC will prepare minutes of their meetings. The minutes will describe the proceedings at the meetings, including a listing of recommendations by the DSMC. Because the minutes may contain unblinded information, it is important that they are not made available to anyone outside the DSMC.

8. Recommendations to the steering group

After the interim analysis meeting, the DSMC will recommend that the steering group continue, hold or terminate the trial. This recommendation will be based primarily on safety and efficacy considerations and guided by statistical monitoring guidelines defined in this Charter and the trial protocol. Recommendations to amend the protocol or conduct of the trial made by the DSMC will be considered and accepted or rejected by the steering group. The steering group will decide whether to continue, hold or terminate the trial based on the DSMC recommendations. The DSMC will be notified of all trial protocol or conduct changes.

9. Statistical monitoring guidelines

The outcome parameters are defined in the REDUSE trial protocol. For the two groups, the DSMC will evaluate data on:

- The primary outcome measure - all cause mortality at 90 days
- The secondary outcome measures
  - Cognitive function measured using the Montreal Cognitive Assessment - BLIND (MoCA-BLIND) at 6 months
  - Health-Related Quality of Life using the European Quality of Life visual analogue scale (EQ-VAS) at 6 months.
  - Days alive and free of invasive ventilation within 90 days of inclusion.
  - Complications in the ICU (cerebral, cardiac, intestinal or limb ischemia or new onset severe AKI).

- Selected exploratory outcomes
  - Hypernatraemia (> 159 mmol/L)
  - Acid base disturbances (hyperchloraemic acidosis [pH < 7.15 and plasma Cl- > 115], Metabolic alkalosis [pH > 7.59 and S-BE > 9]).
  - Central venous catheter related complications that could potentially be related to concentrated drugs given in the intervention group (for example, thrombosis, stenosis, malfunction, and infection
  - Severe hypoglycaemia (≤2.2 mmol/l)

- Suspected unexpected serious adverse events (SUSAEs)

The DSMC will also be provided with these data.

a. Number of patients randomised per intervention group (A, B) per site.
b. Monitoring reports from respective centres

Based on evaluations of these data, the DSMC will decide if they want further data from the steering group and when next to perform analyses of the data. For the interim analysis, the data will be provided in one file as described below. The DMSC will use Lan-DeMets sequential monitoring boundaries, at the all interim analyses of the primary outcome measure, secondary outcomes. The DSMC will also assess if protocol specified event rates

are accurate. If not, the DSMC may suggest an adjustment of trial sample size or duration of follow-up to maintain power. The DSMC should be informed about all SUSACS occurring in the two groups of the trial within 3 days.

10. Conditions for transfer of data from the steering group to the DSMC

The DSMC shall be provided with the data described below in one file The DSMC will be provided with an Excel database containing the data defined as follows:

- Row 1 contains the names of the variables (to be defined below).
- Row 2 to N (where N-1 is the number of patients who have entered the trial) each contains the data of one patient.
- Column 1 to p (where p is the number of variables to be defined below) each contains in row 1 the name of a variable and in the next N rows the values of this variable.

The values of the following variables should be included in the database:
A. PtID: a number that uniquely identifies the patient.
B. Rdcode: The randomisation code (group A or B). The DSMC is not to be informed on what intervention the groups received.
C. 1. EndInd: Primary outcome measure indicator (1 if patient fulfilled the primary outcome measure at day 90 and 0 if the patient did not).
D. MoCA-BlindInd: Montreal Cognitive Assessment score at 6 months
E. EQ-VASLInd: Health-Related Quality of Life at 6 months
F. DAFInd: Day alive and free of mechanical ventilation
G. ComplicationInd: Complications (1 if patient has had a complication as defined above during ICU stay and 0 if the patient did not).
E. ExplorInd: Selected exploratory outcomes as defined above and SUSACS (1 if patient has had an exploratory outcome or SUSAC during ICU stay and 0 if the patient did not).
F. GluInd. Severe hypoglycaemia as defined above

Appendix C. Treatment algorithm for non-resuscitation fluids in the intervention arm.

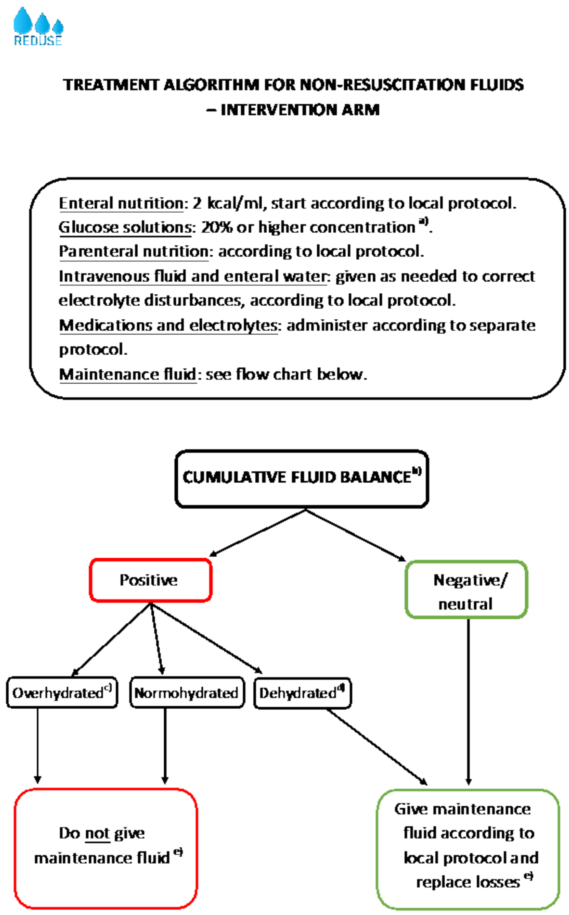

[a] Administer ONLY in patients where enteral nutrition is not tolerated, earliest start 72 hrs after randomization. Maximum dose 1g/kg/day. Glucose solutions can be used earlier in patients at risk for hypoglycaemia (blood glucose < 5 mmol/l and trending downward).

[b] Measured ins and outs:
Ins: nutrition, maintenance fluids, medications and electrolytes, blood transfusions, colloids.
Outs: diuresis, fluid removal from renal replacement therapy, tube drainage, vomiting/gastric tube drainage, bleeding, contents from faecal management system.

[c] Overhydrated (increased total body water relative baseline) as suggested by weight above baseline/preadmission body weight, and/or peripheral/radiological oedema

[d] Dehydrated (decreased total body water relative baseline) as suggested by body weight below baseline/preadmission body weight, decreased skin turgor and/or dry mucus membranes.

[e] Maintenance fluid: intravenous fluid or enteral water prescribed to ensure that total volume of fluid covers basic need of water (approx 1 ml/kg/h).

Appendix D. Medications in the intervention group.

The concentrated solutions should only be used once the patient has a central line. To avoid waste of drug, apply protocol when it´s time to change syringe. Glucose may also be supplied as a vehicle for medication rather than a separate infusion of 20% glucose in patients receiving glucose for the indications described in the treatment algorithm (Appendix C). More concentrated solutions and higher infusion rates than those described below are allowed if already in use at trial site. Drugs not included in the table below should be used in the most concentrated dilution already in use at trial site**.**

Drug	Conc. in stem solution	Suggested dilution in intervention group	Reference	Comments
**Suggested dilutions may only be used if patient has a central line**				
**Vasoactive drugs**				
**Adrenaline**	1 mg/ml	Start at 80 µg/ml and change to 160 µg/ml if infusion rate >10 ml/h	SPC, IM, Micromedex, Halmstad, Borås, UKCPA	**IM;** IV inf 40 - 320 µg/ml, diluted in G. **Halmstad**; 80 µg/ml i NaCl. **UKCPA**; Up to 500ug/ml has been used
**Amiodarone (Cordarone, Amiodaron Hameln)**	50 mg/ml	Dilute to 15mg/ml (20 ml G per 300mg amiodarone)<br><br>Note!<br>Dilute according to local guidelines in cardiac arrest,	SPC, ePED, UKCPA	**SPC**; Dilute in 5% G. **ePed;** 15mg/ml as infusion. **UKCPA**; Many centres infuse daily dose (up to 900mg) in a total volume of 48-50ml
**Dobutamine (Hameln)**	12,5 mg/ml	10 mg/ml	SPC, IM, UKCPA	**IM**; Fluid restr. Adult 2 amps (2x20 ml) + 10 ml NaCl/G give 10 mg/ml.
**Isoprenaline**	0,2 mg/ml	use according to local protocol	Micromedex, Gahart´s, UKCPA	**Halmstad**; 10 ml 0,2 mg/ml in 40 ml G, gives 40 µg/ml. **Micromedex och Gahart´s**; recommend 20 ug/ml for iv bolus and 2 to 4 ug/ml for infusion.
**Milrinone**	1mg/ml	use according to local protocol	SPC	**SPC dilute to 200 ug/ml using G/NaCl.**
**Nitroglycerin (Abcur, BioPhausia)**	1 mg/ml	use according to local protocol	SPC, IM	**SPC**; May be given udiluted using a pump. Can be diluted in G/NaCl. **IM**; 1 mg/ml may be given undiluted

Drug	Conc. in stem solution	Suggested dilution in intervention group	Reference	Comments
**Noradrenaline (Abcur, Pfizer)**	1 mg/ml	Start at 80 µg/ml and change to 160 µg/ml if infusion rate >10 ml/h	SPC, IM, Micromedex, Stabilis	**SPC**; Noradrenaline 1 mg/ml should be diluted with, G/NaCl before use. **IM**; 160 µg/ml. **Micromedex**; G may protect against oxidation. **Stabilis**; 0.5 mg/ml Norepinephrine bitartrate is stabile in G for 48 h at 20-25 °C.
**Levosimendan**	2.5 mg/ml	0.05 mg/ml (5 ml levosimendan 2,5 mg/ml in 250 ml G	SPC 161214, IM	
**Phenylefrine (Abcur, Unimedic)**	0.1 mg/ml	use according to local protocol	Micromedex, IM	**Micromedex**; for iv bolus use 100 µg/ml and 20 µg/ml for inf.
**Vasopressin/ Argipressin (Empressin)**	20 IE/ml	0.4 E/ml	IM, UKCPA	**IM;** 1 amp. (1 ml, 20 units in 50 ml med G, will give conc 0.4 units/ml. **Gahart´s; 1 E/ml**
**Antibiotiotics**				
**Acyclovir**	25mg/ml	25 mg/ml	SPC, UKCPA	**UKCPA;** 25mg/ml over 1 hour by controlled rate infusion. If diluted 5mg/ml infused over at least 1 hour**. SPC; Baxter** Acyclovir 25 mg/ml concentrate for solution for infusion may be administered by a controlled-rate infusion pump.
**Ampicillin**		1 g in 10 ml of sterile water<br><br>2 g in 20 ml of sterile water	SPC, Micromedex	**SPC;** For iv inj. 10 and 20 ml for 1 and 2 g, respectively. **Micromedex;** 1 and 2 g may be diluted in 7.4 and 14.8 ml sterile water, respectively and given in 10-15 min tom minimize risk of seizures. **Meda/Mylan**. Give slowly (minimum 3-4 minutes).
**Anidulafungin**		100 mg in 30 ml of sterile water and add to 100 ml G/NaCl.	SPC, Stabilis, Micromedex, Gahart´s	Infusion rate 1,4 ml/min resulting a total infusion time of 90 min.

**Drug**	**Conc. in stem solution**	**Suggested dilution in intervention group**	**Reference**	**Comments**
**Bensylpenicillin**		1 g in 10 ml of sterile water<br><br>3 g in 20 ml of sterile water	SPC, IM	**SPC; dissolve** 1 g in 10 ml of sterile water and 3 g in 20-40 ml of sterile water. **IM;** 600 mg in 4-10 ml. Inject slowly (> 3-5 minutes)
**Caspofungin (Cancidas)**	50 mg<br><br>70mg	Carefully dissolve 50 mg in 10,5 ml of sterile water. Add to 100 ml NaCl.<br><br>Carefully dissolve 70 mg l in 10,5 ml of sterile water. Add to 140 ml NaCl	Micromedex, SPC, IM	Give drug during at least 60 min!
**Cefotaxime (Sandoz, Stragen, Villerton)**		1 g in 4 ml sterile water<br><br>2 g in 10 ml sterile water	SPC	**SPC Sandoz**;Note that rapid injection of cefotaxim in central line has been reported to cause life threatening arrythmia in rare cases.
**Ceftazidime (Fortum)**		1 g in 10 ml of sterile water<br>2 g in 10 ml of sterile water	SPC, IM, Micromedex	
**Ceftriaxone (Fresenius Kabi)**		1 g in 10 ml of sterile water<br>2 g in 20 ml of sterile water	SPC , Stabilis, SPC Stragen, IM. Micromedex	**Fresenius Kabi**, Use NaCl or G for 2 g. **Stabilis;** 100 mg/ml in water is ok. **Micromedex;** 2 g in 10 ml. **IM;** Infusion if dose ≥ 2 g.
**Cefuroxime (C.Stragen, Zinacef)**		750 mg in 6 ml of sterile water<br><br>1,5 g in 15 ml of sterile water	SPC, IM	
**Clindamycine (Dalacin)**	150 mg/ml	600 mg in 50 ml G/NaCl (gives 11 mg/ml)	SPC, IM	**IM;** Final concentration max 18 mg/ml. **SPC, IM;** shortest infusion time is 600 mg in 20 min.
**Cloxacillin (C. Stragen)**		1 g in 20 ml of sterile water<br><br>2 g in 40 ml of sterile water	SPC	**Stabilis** konc up to 250 mg/ml are ok.

Drug	Conc. in stem solution	Suggested dilution in intervention group	Reference	Comments
**Doxycycline (Doxyferm)**	20 mg/ml	100 mg in 100 ml G/NaCl<br><br>200 mg in 200 ml G/NaCl	SPC	
**Erythromycin**		1 g in 20 ml sterile water and add 80 NaCl.	IM, ePed, SPC,	**IM**; Final concentration should not be greater than 10 mg/ml. **ePed;** Give dose in > 1h to mimimize risk of arrythmias.
**Gentamycin (Gensumycin)**	40 mg/ml	May be given undiluted as bolus.<br><br>Repeated doses either diluted or as boluses over 3-5 minutes depending on dosing regimen.	SPC, UKCPA	**SPC**; If administered twice daily gentamycin may be given undiluted in 3-5 minutes. **UKCPA**; For large doses i.e., most centres dilute with 50 ml G/NaCl.
**Imipenem/Cilastatin (Tienam, I/C Fresenius Kabi)**		500/500 mg in 10 ml NaCl and add to 90 ml NaCl/G. Maximum concentration of imipenem 5 mg/ml	IM, SPC, UKCPA	**SPC;** doses ≤ 500 mg/500 mg should be given over 20 to 30 minutes and doses >500 mg/500 mg should be given over 40 to 60 minutes.
**Meropenem**		For bolus dilute in sterile water to a final concentration of 100mg/ml<br><br>For 3h infusion dilute to 50 mg/ml with sterile water or NaCl<br><br>For 8-12h infusions dilute to 20mg/ml with sterile water or NaCl	IM, SPC, UKCPA, ePED, Lexicomp	**IM;** 0.5-1 g doses in 5 min. 2 g doses in 15-30 min. **Lexicomp/ePED** Meropenem diluted in sterile water/NaCl to 50 mg/ml is stable for 3h in room temperature. **Barton;** Meropenem for 8-12h infusions should be diluted to 20-40mg/ml
**Metronidazole (Braun)**	5 mg/ml	Undiluted		
**Piperacillin/Tazobactam**		2/0,25 g in 10 ml of sterile water/NaCl	SPC, IM,	

Drug	Conc. in stem solution	Suggested dilution in intervention group	Reference	Comments
		4/0,5 g in 20 ml of sterile water/NaCl<br><br>For infusions dilute further with G/NaCl to 50ml		
**Tobramycin (Nebcina)**	40 mg/ml	Use undiluted	IM, SPC	
**Tobramycin (Nebcina)**	80 mg/ml	80 mg/ml dilute with 50 ml G/NaCl	SPC	**SPC;** shorter infusion time than 20 minutes will increase risk for toxic side-effects and is not recommended.
**Trimethoprim/Sulfamethoxazole (EUs prim)**	16+80 mg/ml (5 ml/amps)	2 amps. in 150 ml G.<br>Observe carefully for precipitates.<br><br>4 amps. in 300 ml G	SPC, IM (Co-trimoxazole)	**SPC;** Stable for 2 h! **IM;** possible to give undiluted stock solution in 60-90 min (off label).
**Vancomycin (Orion)**		500 mg in 10 ml sterile water. Add to 40 ml NaCl/G to give a conc. of 10 mg/ml<br><br>1 g in 20 ml of sterile water. Add to 80 ml NaCl/G to give a conc. of 10 mg/ml	IM, UKCPA	**IM;** In exceptional circumstances 20 mg/ml may be given via a central line. **UKCPA;** 10mg/ml is a commonly used dilution. 20mg/ml has been used in some centres. **IM;** give in 1 h. **Regional dilution routine;** Give a dose of 500 mg in 60 min and 1g in a 100 min.
**Voriconazole (Vfend)**	200 mg	200 mg in 19 ml of sterile water to a conc. of 10 mg/ml. For doses 50-500 mg: add to 100 ml G/NaCl. For doses > 500 mg: add to 250 ml G/NaCl.	IM, SPC	Final concentration should be 0,5-5,0 mg/ml. Max infusion rate is 3 mg/kg/h.
**Fluconazole**	2 mg/ml	Use undiluted	SPC	Infusion rate 10 ml/min or lower.
**Other drugs**				

Drug	Conc. in stem solution	Suggested dilution in intervention group	Reference	Comments
**Clonidine (Catapresan)**	150 µg/ml (1 ml ampule)	30 µg/ml	SPC, IM, UKCPA	**UKCPA;** 6-50 micrograms/ml infusion. Diluent: Sodium chloride 0.9% or glucose 5%.
**Dexmedetomidine**	100ug/ml	8ug/ml	SPC	
**Insulin, human (Humulin Regular, Actrapid)**	Insulin, human (Humulin Regular)	1 E /ml	Stabilis	**Stabilis;** Dilute in NaCl
**Levetiracetam**	100 mg/ml	250-1500 mg in 100 ml NaCl/G, give in no less than 15 min.	SPC, IM, Micromedex, Gahart´s	**Micromedex;** Do not exceed a final max cons of 15 mg/ml. Can be given as iv bolus, 3-5 min and continuous infusion 200-400 mg/h.
**Magnesium sulphate (**Addex-Mg)	1 mmol/ml	0.5 mmol/ml, (20 ml in 20 ml NaCl, giving a conc. 0,5 mmol/ml). Give in no less than 10 min.	IM, Gahart´s, VGR guideline, UKAP	**Gahart´s;** D5W and NS are the most common diluents. **UKCPA**; suggested dilutions 1-2mmol/ml
**Potassium hydroxide/ Potassium phosphate (Addex-Kalium)**	2 mmol/ml	1-2 mmol/ml dilute in NaCl if needed	SPC	**SPC;** Give at most 20 mmol potassium/h.
**Potassium Chloride**	2 mmol/ml	1-2 mmol/ml, in NaCl dilute if needed	SPC	**SPC;** Give at most 20 mmol potassium/h.
**Propofol (Propofol-Lipuro)**	10 or 20 mg/ml	20 mg/ml for infusion. According to local routine for intubation	SPC	
**Sodium glycerophosphate (Glycophos)**	1 mmol/ml	0.5 mmol/ml 20 ml sodium glycerophosphate 1mmol/ml in 20 ml NaCl.	ePED	**ePED**; Administer in no less than 8 h.

Drug	Conc. in stem solution	Suggested dilution in intervention group	Reference	Comments
**Thiamine**	50mg/ml<br>125mg/ml	50 mg/ml, give in no less than 30 min	ePED, Micromedex	**ePED**; Can be diluted with NaCl/G to achieve suggested dilution. Lower doses may be diluted to a total volume of 10 ml to facilitate administration.

NaCl = Sodium chloride 9 mg/ml = NS, **G=**Glucose 50 mg/ml=Dextrose 5%=D5W

Barton= Barton G, Rickard CM, Roberts JA. Continuous infusion of beta-lactam antibiotics in critically ill patients with sepsis: implementation considerations. Intensive Care Med. 2024; 50: 2150-2153. doi: 10.1007/s00134-024-07650-x. **ePED =** experience and evidence-based database for paediatric medicines (https://eped.se/about-eped/) accessed January 31, 2024.**Gahart´s** = Gahart´s 2021 intravenous medication via https://www.clinicalkey.com. **IM** = UCL Hospitals Injectable Medicines Administration Guide: Pharmacy Department, 3rd Edition, University College London Hospitals, ISBN: 978-1-405-19192-0, **Lexicomp =** Lexicomp Online, Waltham, MA: UpToDate, Inc. Accessed January 31, 2024. **Micromedex** = https://www.micromedexsolutions.com, **Halmstad** = vårdriktlinje "Inotropa läkemedel och vasopressorer HSH" published in 2020-09-13, **Regional dilution routine Region Skåne, Sweden** = www.lakemedelshantering.se, **Stabilis** = https://www.stabilis.org, **SPC** = summary of product characteristics, **UKCPA** = United Kingdom Clinical Pharmacy Association, Minimum infusion volumes for fluid restricted critically ill patients, v 4.4 2012

Appendix E. Model patient information and informed consent form

Research subject information for you who were treated in the intensive care unit for septic shock regarding the study:

Can protocol-directed administration of fluids given for purposes other than circulatory stabilization improve outcome in septic shock?

Entity responsible for research: Region Skåne

Principal investigator: Peter Bentzer, Professor, Senior Consultant
Charlotte Yhléns gata 10
252 23 Helsingborg.
Telephone number: 042 - 4061000.

You have been admitted to an intensive care unit and treated for septic shock, a condition caused by an infection in the body. In cases of septic shock, it is common to receive large amounts of fluid. Some of the fluid is given to maintain sufficient blood volume, while some may be given, for example, together with medications or as nutrition. Fluid administration can be life-saving, but research suggests that too much fluid can be harmful. We are therefore carrying out an investigation to see if we can improve the course of care and prognosis for you as a patient by reducing how much fluid is given.

We have let chance determine whether you received a "normal" amount of fluid or a reduced amount of fluid. Since you were very ill, we could not ask if you wanted to participate in the study until now, but we have consulted with your relatives and informed them about your participation in the study.

We have collected information about your care in the intensive care unit and up to 36 ml of blood for future chemical analyses.

To study whether the differences in treatment between the two groups affect recovery over time, we will contact you by phone about 6 months after you were admitted to the intensive care unit. During this call, we will ask you about your health and how you manage your daily activities. You will also be asked to take a memory test. The call takes just over an hour. If you prefer to visit the hospital, this can be arranged. You are welcome to bring a close friend/relative if you wish. Travel expenses will be reimbursed for both of you.

It is very important for the study that as many people as possible participate in the follow-up, regardless of whether you feel well or unwell. The tests we use to collect information about your recovery have been used in many other studies and can detect even small issues (such as memory problems) that can affect your recovery and daily life. If we find that you have

persistent problems, we will ask if you feel you have received the help you needed. If not, we will refer you to an appropriate specialist such as an occupational therapist, physiotherapist, psychologist, neurologist, rehabilitation doctor, or general practitioner for further examination, advice, and support.

All data collected for the study is stored in an electronic database that meets all confidentiality requirements to protect your privacy. When you were included in the study, you received a study code. All data and samples collected as part of the study are stored under this code. You can only be linked to your study code via a code list kept in a locked space at the hospital where you were treated. Data is stored for 15 years and no unauthorized person will have access to it. Anonymized data may be shared with foreign researchers.

Participation in the study is voluntary and you can decline participation at any time. Further data collection will then be stopped. In this situation, we will ask if we can use data that has already been collected. If you do not consent, this data will be deleted. You also have the right to see what information has been collected and to have any inaccuracies corrected. You are welcome to contact the responsible researchers listed below at any time if you have questions about the study. If you are dissatisfied with the way your personal data is processed, you have the right to file a complaint with the Swedish Data Protection Authority (Integritetsskyddsmyndigheten), which is the supervisory authority for this study.

The blood samples collected will be stored in a "biobank" and the samples will be coded as described above. We intend to use the biobank to find biochemical markers that can help us predict the response to treatment and to map mechanisms behind any effect of the different treatments. We will not conduct any genetic analyses. The samples will primarily be analyzed in Sweden but may also be analyzed abroad. After analysis, the samples will be destroyed. The samples will be handled according to the biobank law (2023:38).

If you choose to withdraw from the study, you should contact the principal investigator, who will then ensure that the samples collected from you are destroyed. In this situation, we will ask if we can use sample results that were already collected. If you do not consent, such results will be deleted. If additional research is planned in the future, the Swedish Ethics Review Authority (Etikprövningsmyndigheten) will decide whether you should be asked again. The principal investigator may be contacted if you want more information about the biobank.

Region Skåne (organization number 232100-0255) is responsible for your personal data according to the General Data Protection Regulation (GDPR). For questions about how data is handled under the GDPR, you can contact:

Personuppgiftsombudet i Region Skåne, 291 89 Kristianstad.

Telephone: 044-309 30 00; E-mail: region@skane.se

Patient indemnity insurance applies to this examination.

Those locally responsible for the REDUSE study are:

City, 2020-xx-xx

NN, Job Title

The Clinic for Anesthesia and Intensive Care

XXX XX, Name of hospital
Telephone:
E-mail:

CONSENT FORM

Can protocol-directed administration of fluids given for purposes other than circulatory stabilization improve outcome in septic shock?

I have been informed about the study both orally and through a written patient information sheet.

I believe that I have had the opportunity to ask questions and that I have had them answered.

I agree to participate in the above study and to my personal data being stored.

I also consent to have the information in my patient record reviewed to see if it matches the information stored in the study database. This review will be conducted by an external study monitor (examiner) to ensure the quality of the study.

I consent to my blood samples being stored in a biobank and that they will be analyzed for purposes described in the information above.

Patient

Location: .... Date: ....

Signature: ....

Name clarification: ....
